# HIV infection reprogrammes CD4+ T cells for quiescence and entry into proviral latency

**DOI:** 10.1038/s41564-025-02128-y

**Published:** 2025-09-26

**Authors:** Leah M. Plasek Hegde, Lalith S. Gunawardane, Farshad Niazi, Uri Mbonye, Konstantin Leskov, Gani Perez, Curtis Dobrowolski, Meenakshi Shukla, William S. Nutt, Jonathan Karn, Saba Valadkhan

**Affiliations:** 1 Department of Molecular Biology and Microbiology, Case Western Reserve University School of Medicine, Cleveland, OH USA; 2 Present Address: Department of Molecular, Cellular and Developmental Biology, Yale University, New Haven, CT USA; 3 Present Address: Section of Molecular Neurogenetics, Medical Genetics Branch, National Human Genome Research Institute, National Institutes of Health, Bethesda, MD USA; 4 Present Address: Department of Biomedical Engineering, Georgia Institute of Technology, Atlanta, GA USA; 5 Present Address: General Clinical Studies Unit, Mayo Clinic, Jacksonville, FL USA; 6 Present Address: Molecular and Cellular Biology Program, University of Washington, Seattle, WA USA

**Keywords:** Virus-host interactions, Viral reservoirs

## Abstract

Human immunodeficiency virus (HIV) persists in infected individuals despite effective antiretroviral therapy due to the rapid establishment of latent reservoirs, mainly composed of quiescent memory CD4+ T cells. The mechanisms governing latent reservoir formation remain poorly understood. Here, using single-cell RNA-seq and functional studies in human primary CD4+ T cell models, we show that HIV infection with reporter constructs and laboratory and patient-derived strains triggers transcriptomic remodelling, activating the p53 pathway and a quiescence programme mediated by Krüppel-like factor 2 (KLF2), a key quiescence regulator. Loss- and gain-of-function studies, including unbiased shRNA screens and confirmatory studies in CD4+ T cells from HIV+ donors, demonstrate that HIV infection drives KLF2 and p53 signalling, which downregulate MYC and proliferation pathways, resulting in proviral transcriptional silencing. This enhances latent reservoir formation in T cells, ensuring viral persistence. These findings present a mechanism for forming the latent HIV reservoir and broaden the repertoire of strategies through which viruses control host cells to their advantage.

## Abstract (web summary)

HIV infection triggers transcriptomic remodelling and a quiescence programme via KLF2 and the p53 pathway leading to proviral silencing.

## Main

The cessation of antiretroviral therapy in HIV-infected individuals is almost invariably followed within weeks by a return of viraemia due to the activation of a persistent and latent reservoir of HIV-infected cells^1–3^. Eradication of these latently infected cells, which largely consist of memory CD4+ T cells, microglia and, to a smaller extent, macrophages, requires the development of novel therapeutic strategies^4–6^. Most of the latent reservoir is found in quiescent CD4+ memory T cells^7–9^. Recent studies have shown that during the transition from proliferating effector to quiescent memory cells, remodelling of the cellular gene expression programme facilitates the entry of an integrated provirus to latency^7–11^. While entry into quiescence is thought to play a dominant role in the induction of proviral latency, additional mechanisms, such as direct infection of resting cells^7,9,12^, the genomic locus of the provirus^7,9,13–15^, stochastic fluctuations in the expression of the viral Tat protein and cellular proteins such as KAP1 (refs. ^16–18^) also help regulate proviral entry into latency.

The mechanisms involved in entry of HIV-infected cells into quiescence and their impact on proviral transcription remains largely unstudied, partly because this requires modelling in realistic primary cell models. Several factors have been implicated in the induction and maintenance of quiescence in uninfected cells, including key regulatory factors KLF2 (Kruppel-like factor 2), a zinc finger transcription factor^19–21^, and the p53-induced antiproliferative factor TOB1 (Transducer of ERBB2-1)^22^. Both proteins are known to be highly expressed in quiescent cells, and upon activation of T cells, their cellular levels are strongly downregulated to nearly undetectable levels^20–22^; however, direct evidence that they play a regulatory role in primary human T cells is lacking. TOB1 appears to partly mediate the cell cycle arrest caused by activation of p53 (ref. ^23^). Overexpression of KLF2 in human CD4+ T cell lines and in vivo in the mouse led to a marked downregulation of proliferation and MYC expression, followed by cellular quiescence characterized by inhibition of proliferation, decreased cell size and reduction in cellular activation markers^20,21,24^. Similarly, KLF2 loss-of-function studies in vitro and in vivo in the mouse CD4+ T cells led to spontaneous entry into the S phase, an activated phenotype with increased cell size and expression of proliferation markers and a high level of apoptotic death that mimicked activation-induced cell death^20,25^. However, some of these observations could be attributed to the essential role of KLF2 for mature T cell egress from the thymus^24,26^. The pro-quiescence impact of KLF2 could be rescued by the expression of MYC in Jurkat cells^20,21^. Further, expression of a dominant negative MadMyc fusion protein could largely recapitulate the impact of KLF2 overexpression in Jurkat cells^20^. Together these results indicate that KLF2 is a powerful inducer of quiescence in CD4+ T cells acting at least partly through downregulation of MYC expression. However, key questions such as whether the same factors are involved in induction of quiescence in infected cells and the conditions in which the quiescence programme is activated in productively infected cells remain unanswered. Whether KLF2 plays a similar role in CD8+ T cells remains unstudied^27,28^.

## Results

**Fig. 1:**
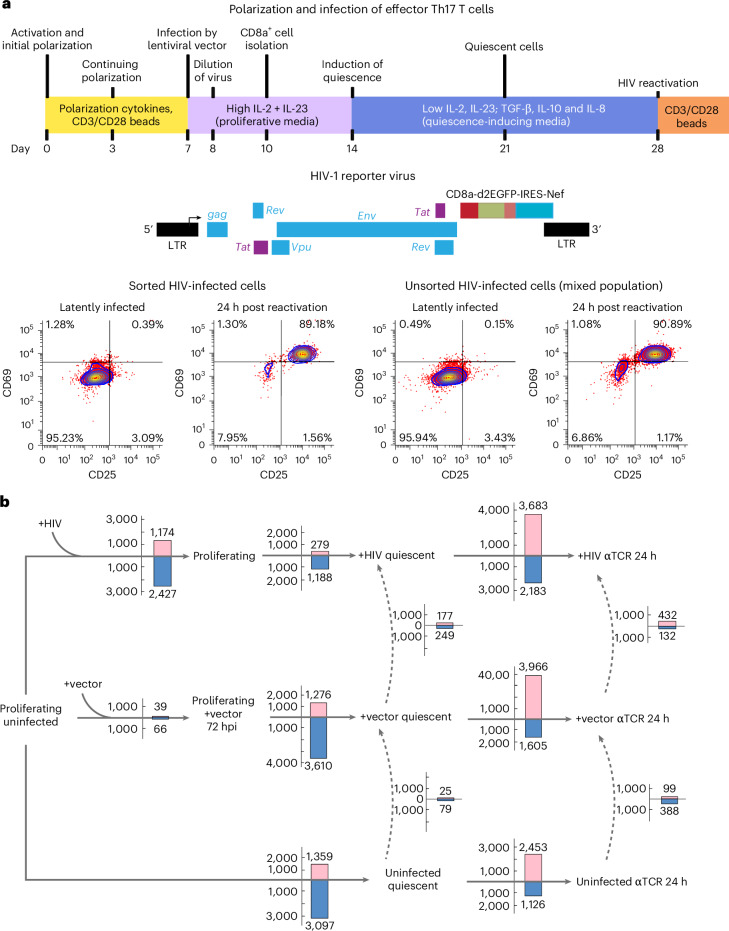
HIV infection induces a strong pattern of transcriptional shutdown. **a**, Schematic representation of the timecourse of ex vivo CD4+ T cell HIV latency QUECEL model and HIV reporter construct used in the majority of experiments in this study. In the HIV reporter construct schematic, the location of each gene is shown, along with the locus of the CD8a-EGFP-Nef fusion reporter gene. Representative flow cytometry analyses are included, each containing two panels corresponding to days 27 and 29 of the QUECEL timecourse. **b**, HIV-infected cells require markedly fewer transcriptomic changes to enter the quiescent state compared with identically maintained uninfected or vector-infected cells. The number of genes up- or downregulated during transition between different stages of HIV and T cell life cycle are shown. Pink and blue bars reflect the number of up and downregulated protein-coding genes during each transition or comparison, respectively. Dashed vertical lines compare the gene expression patterns of uninfected (untreated HIV-naive), vector-infected and purified HIV-infected cells after achieving full quiescence, or 24 h after TCR stimulation, showing that the 3 experimental groups achieve nearly identical transcriptomic patterns in either the quiescent or reactivated state. Source data

We analysed global gene expression changes during the HIV life cycle using ex vivo latency models of primary human CD4+ T cells (QUECEL), which provide highly pure, homogeneous populations of Th1, Th2, Treg and Th17 polarized, HIV-infected cells. The polarized cells were infected using a pseudotyped, single-round HIV virus based on the NL4-3 strain containing a CD8-EGFP fusion protein (Fig. 1a), which can be used to gently and efficiently purify the infected cells^11,29^. The QUECEL model has been extensively characterized by imaging of proviruses from infection through quiescence^30^ and measurements of the sequestration of key transcription factors^31^ and heterochromatin during quiescence^32^.

**Fig. 2:**
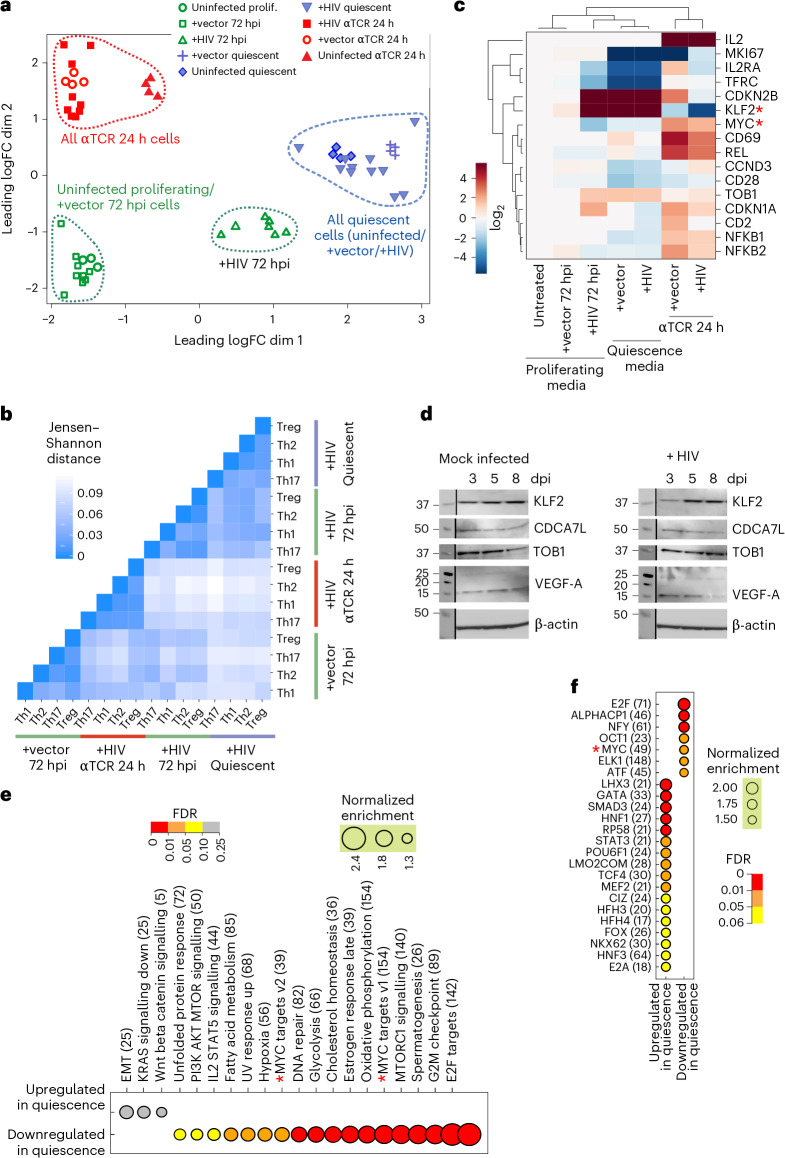
HIV infection, entry into and exit from quiescence are accompanied by strong changes in overall gene expression patterns. **a**, Dimensionality reduction study using multidimensional scaling indicates the presence of strong transcriptomic changes in early timepoints following HIV infection. **b**, Jensen–Shannon distance analysis of the overall transcriptomic pattern of the four polarized QUECEL ex vivo models at each step of the HIV life cycle indicates strong similarities between the quiescent and 72-hpi HIV-infected cells. **c**, Expression of quiescence and proliferation markers at different stages of HIV and T cell life cycle points to the induction of a quiescence-like pattern 72 h following HIV infection resembling that seen in cells maintained in quiescence-inducing media for 2 weeks (cells labelled ‘Quiescence media’). Asterisks mark MYC and KLF2, key factors in regulation of T cell activated and quiescent states, respectively. **d**, Western blots indicating the timecourse of changes in expression of KLF2, TOB1 and two MYC-regulated effectors CDCA7L and VEGF-A at the protein level, which corroborate the pro-quiescence transcriptomic patterns observed in **c** following HIV infection. Molecular weight markers are shown on the left in kilodaltons (kDa). This western blot experiment was performed twice independently with similar results. dpi, days post infection. **e**, Several pathways from the Hallmark gene lists of mSigDB, including MYC signalling (marked by asterisks), were significantly positively and negatively enriched during transition of vector-infected and uninfected cells from proliferating to quiescent state. Numbers in parentheses indicate the number of genes in each pathway that drive the enrichment phenotype (see Methods). **f**, The activity of multiple transcription factors, including MYC (marked by an asterisk), show strong changes after entry into quiescence in uninfected and +vector primary CD4+ T cells. Source data

**Extended Data Fig. 1:**
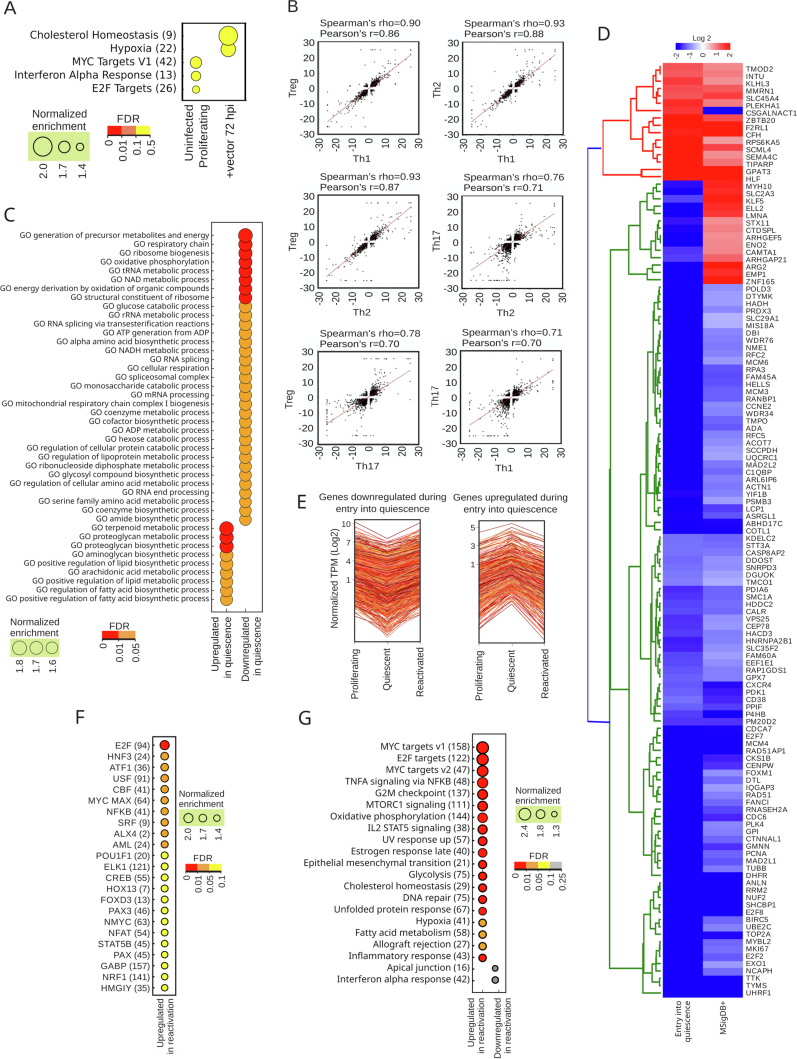
HIV infection leads to a strong downregulation of metabolic pathways. **a**. Pathway analysis comparing uninfected proliferating, and +vector 72 hpi cells indicates mild to moderate changes in a small number of pathways with marginal statistical significance. Numbers between parentheses indicate the number of genes that drive the enrichment phenotype (see Methods). **b**. Pairwise comparison of the pattern of differential expression of protein-coding genes in response to HIV infection between the four polarized effector cells show pairwise correlation coefficients of 0.7-0.88. Scatterplots show genes differentially expressed 72 hpi in pairs of polarized cell ( > 2-fold change, FDR < 0.05). **c**. Transition into quiescence leads to downregulation of cellular metabolic pathways. The metabolic process subset of Gene Ontology (GO) pathways that are differentially enriched in either the positive or negative direction during transition from proliferative state to quiescence in vector-infected cells with an FDR < 0.05 were included in the graph. **d**. Genes showing differential expression (defined as >2-fold change with FDR < 0.05) during transition from proliferating to quiescent state in vector-infected cells closely match the genes in quiescence pathways in mSigDB and other studies of quiescence (mSigDB + , see Methods). **e**. A large fraction of protein-coding genes show symmetric changes in expression during entry into and exit from quiescence. Each line corresponds to the pattern of gene expression of a single differentially expressed gene (showing >2-fold change with FDR < 0.05 during both transitions). **f**. and **g**. Transcription factors (**f**) and pathways (**g**) that show altered activity or enrichment in uninfected and +vector αTCR 24 h cells compared to quiescent cells. Numbers between parentheses indicate the number of genes in each gene list that drive the enrichment phenotype (see Methods). Source data

We performed RNA-seq on untreated, vector-infected (+vector) and purified HIV-infected (+HIV) cells in proliferation-supporting media 72 h post infection (72 hpi); 2 weeks after the addition of quiescence-inducing cytokines when full quiescence and proviral latency are achieved; and 24 h after reactivation through T cell receptor (TCR) stimulation (αTCR 24 h) (Fig. 1a)^11^. Differential expression tests and dimensionality reduction studies indicated that within the quiescent and αTCR 24-h groups, the uninfected, +vector and +HIV cells clustered together and have very similar global gene expression patterns (Figs. 1b and 2a), which is consistent with published data^33,34^. Dimensionality reduction, differential expression studies and pathway analysis on uninfected versus +vector cells maintained in proliferative media indicated that these two cellular populations were nearly identical in gene expression pattern (Figs. 1b and 2a, and Extended Data Fig. 1a). In contrast, +HIV 72 hpi formed a distinct cluster (Fig. 2a), indicating virally induced changes to the transcriptome, which was confirmed by differential gene expression studies (Fig. 1b). Th1, Th2, Th17 and Treg polarized cells showed virtually identical change in gene expression pattern in the above studies regardless of polarization identity (Fig. 2b and Extended Data Fig. 1b).

The use of QUECEL models enabled us to investigate the poorly understood process of entry of primary human CD4+ T cells into quiescence at high transcriptomic resolution. Quiescence state transcriptomic signatures that were shared among uninfected, +vector and +HIV quiescent cells included known pro-quiescence regulatory factors KLF2, TOB1 and CDKN2B (Fig. 2c,d). By contrast, multiple pathways, including MYC signalling and MTORC pathways, both of which are known to be key drivers of the T cell proliferative phase^35^, were downregulated, along with pathways related to cell cycle, transcriptional and translational regulation, and cellular metabolism (Fig. 2e,f and Extended Data Fig. 1c). Similarly, genes known to be up- or downregulated during entry into quiescence, based on mSigDB gene sets and published studies^36^, overwhelmingly showed differential regulation in the expected direction during entry of uninfected cells into quiescence in QUECEL models (Extended Data Fig. 1d), further confirming the quiescent identity of these cells.

These changes in gene expression were largely reversed after TCR stimulation. Most genes downregulated during entry into quiescence were upregulated after reactivation and vice versa (Extended Data Fig. 1e). The majority of genes differentially expressed following TCR stimulation were upregulated, indicating a global rise in transcriptional activity (Fig. 1b). The upregulated genes and pathways included several cellular growth and proliferation markers and pathways, including MYC and E2F signalling, while quiescent markers were downregulated (Fig. 2c and Extended Data Fig. 1f,g). However, several additional pathways, such as TNF and IL2-STAT5 signalling, which were not enriched during entry into quiescence, were among the reactivation-induced gene sets (Extended Data Fig. 1f,g).

### HIV infection leads to transcriptomic changes identical to those observed in quiescent cells

**Fig. 3:**
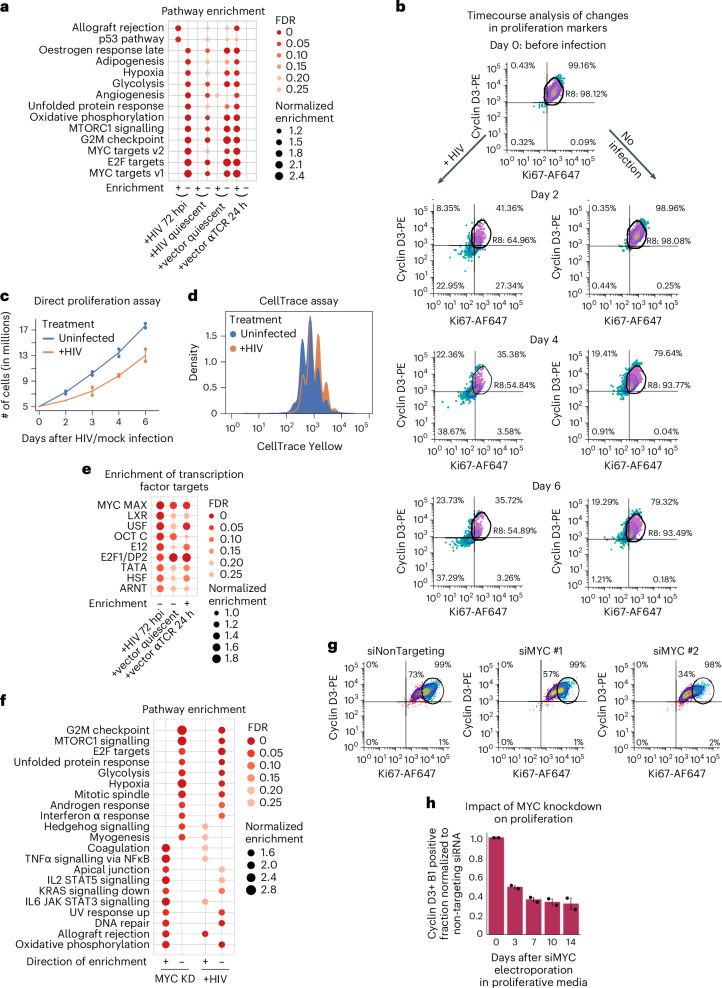
HIV infection leads to strong downregulation of the key proliferation factor MYC and decrease in cellular proliferation. **a**, Several proliferative pathways and gene sets are negatively enriched at 72 hpi. For comparison, pathways changing in +vector cells during entry into and exit from the quiescence state are shown, along with those changing during the transition of +HIV 72-hpi cells to full quiescence. For this panel and in **e** and **f**, plus and minus signs at the bottom indicate positive or negative enrichment, respectively. **b**, Flow cytometry analysis of unpurified, minimally disturbed HIV-infected and identically treated uninfected cells after 2, 4 and 6 days of infection or mock infection indicates a reduction in proliferation markers in HIV-infected cells. The circular gate marks the position of proliferative cells prior to HIV infection or mock infection, with cells inside and outside this gate shown as purple and blue dots, respectively. **c**, HIV infection leads to a slowdown of cellular proliferation. Uninfected and HIV infected cells from 2 healthy donors were grown in proliferative media in the presence of IL-2 and IL-7, and cell numbers for each group were counted at indicated timepoints after infection or mock infection. In this panel and in **h**, values shown are the mean ± s.d. of 2 biological replicates. **d**, CellTrace proliferation assays confirm the slowed proliferation rate of HIV-infected cells. Peaks representing cells with decreasing CellTrace signal mark each round of cell division after the application of CellTrace Yellow stain (see Methods). **e**, Targets of proliferative transcription factors including MYC and E2F1 are negatively enriched 72 h after HIV infection. For comparison, changes in transcription factor activity during entry into and exit from quiescence in +vector cells are shown. **f**, Knockdown of MYC in CD4+ T cells results in a transcriptomic profile closely similar to the one observed 72 h after HIV infection, characterized by the negative enrichment of key proliferative pathways. **g**,**h**, Knockdown of MYC using siRNAs (siMYC) results in strong loss of expression of Ki67 and cyclins D3 and B1 compared to cells treated with a non-targeting siRNA. Both control and knockdown cells obtained from a total of 3 healthy donors (2 donors in **h**) were incubated in proliferative media containing high levels of IL-2. Circular gate in **g** marks the position of proliferative cells, with cells inside and outside the gate shown in blue and red, respectively. Source data

**Extended Data Fig. 2:**
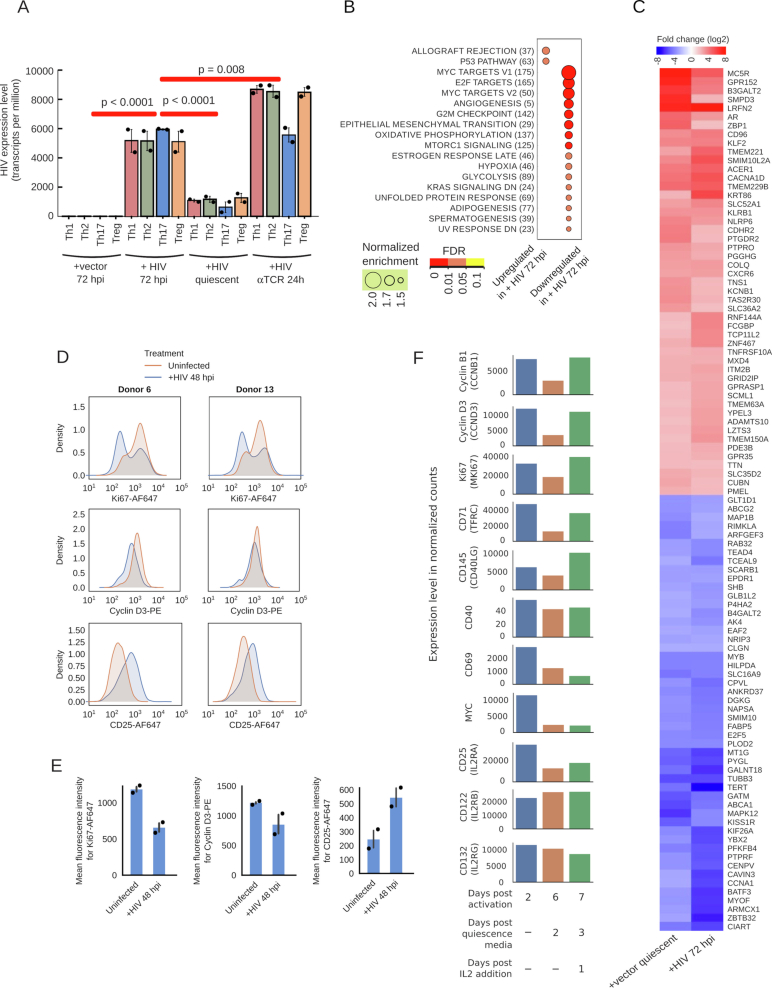
HIV-induced downregulation of metabolic and proliferative pathways does not result from a block to IL2 receptor signaling. **a**. Changes in HIV proviral gene expression in +HIV 72 hpi, +HIV quiescent and +HIV αTCR 24 h conditions in the four main polarized subtypes of effector CD4 + T cells. The Y axis indicates the number of transcripts per million cellular transcripts calculated using bulk RNA-seq studies from two independent healthy donors (with 2-4 technical replicates per polarized identity per donor for some conditions) which are infected/mock infected *ex vivo*. +vector 72 hpi cells are included as negative controls. In this panel and **e**, shown values represent the mean of the biological repeats +/- standard deviation. Statistical comparisons were performed using a linear mixed-effects model with HIV status/timepoint group as a fixed effect, donor as a random intercept, and polarization as a random effect. Relative to the +HIV 72 hpi group as the reference, the αTCR 24h group had a log10(TPM) estimated difference of +0.163 (95% CI: 0.042 to 0.284; Cohen’s *d* = 2.15), the quiescent infected group –0.713 (95% CI: –0.834 to –0.592; *d* = –4.54), and the mock 72 hpi group –2.640 (95% CI: –2.805 to –2.475; d<-10 ). **b**. HIV infection leads to downregulation of multiple cellular growth pathways including MYC and E2F transcriptional cascades and upregulation of p53 signalling. Numbers between parentheses indicate the number of genes in each gene list that drive the enrichment phenotype (see Methods). **c**. A large fraction of genes differentially expressed in +HIV 72 hpi cells compared to +vector 72 hpi and uninfected proliferative cells (2660 of ~3600 genes, Fig. 1b) correspond to the genes differentially expressed during entry into quiescence in the absence of HIV (in +vector and uninfected cells). The top 50 most differentially expressed among these correlated genes in each direction are shown. **d**. Flow cytometry analysis indicates that at 48 hours post-infection, unpurified HIV infected cells show decreased expression of proliferation markers cyclin D3 and Ki67 relative to uninfected cells. However, at this timepoint the infected cells have not yet downregulated the level of IL-2 receptor CD25 found on the cell surface. CD25 protein expression on cell surface is in fact elevated at this early infection time point. **e**. Mean fluorescence intensity of AF647 anti-Ki67, PE anti-cyclin D3, and AF647 anti-CD25 signal detectable in uninfected and *ex vivo* HIV infected cells from two independent healthy donors at 48 hpi in proliferative media. **f**. Despite the reduced level of CD25/IL2RA mRNA after 3 days of exposure to quiescence media, addition of IL2, even at the very low dose of 15 IU/ml (shown here), leads to a strong upregulation of its downstream targets. Importantly, the level of IL2 receptor beta and gamma subunits does not change during entry into quiescence. CD4 + T cells were activated through TCR stimulation, and after 4 days in proliferative media were plated in the quiescence-inducing media for 2 days, followed by the addition of 15 IU/ml IL2 (instead of 60 IU/ml used in proliferative media). Samples were taken from cells 2 days after reactivation, and before and 24 hours after the addition of IL2 and were subjected to bulk RNA-seq. Source data

Unexpectedly, dimensionality reduction studies and Jensen–Shannon divergence calculations (Fig. 2a,b) showed that +HIV 72-hpi cells (Extended Data Fig. 2a) clustered close to quiescent cells. Compared to uninfected and +vector 72-hpi cells maintained in the same proliferation-supporting media, +HIV 72-hpi cells showed a predominant pattern of transcriptional downregulation, with over 1,150 and 2,400 protein-coding genes up and downregulated, respectively (Fig. 1b). The cellular pathways altered after HIV infection (Fig. 3a and Extended Data Fig. 2b) closely resembled those downregulated after quiescence entry in uninfected and +vector cells (Figs. 3a and 2e). Among the ~3,300 protein-coding genes that were differentially expressed in both uninfected and +vector cells when entering quiescence, over 2,650 showed a correlated change in expression in +HIV 72-hpi cells (Extended Data Fig. 2c), further demonstrating strong similarities between quiescence and 72-h post HIV infection gene expression patterns.

The HIV-downregulated pathways correspond to key proliferative pathways, including MYC and mTORC1 signalling, consistent with reduced transcriptional and translational activity, bolstering the above results. Metabolic pathways such as glycolysis, adipogenesis and oxidative phosphorylation similarly showed reduced activity (Fig. 3a and Extended Data Fig. 2b). Pathways regulated by E2F and HIF1A (hypoxia-related pathways), also important players in the T cell proliferative state^37,38^, were likewise downregulated (Fig. 3a and Extended Data Fig. 2b), even though the +HIV 72-hpi cells were maintained in proliferation-supporting media containing a high level of IL-2 (60 IU ml^−1^), a known inducer of proliferation in T cells. Activation of the quiescence transcriptomic programme in HIV-infected cells did not stem from an inability to respond to IL-2, as we could show that despite downregulation of the IL-2 receptor IL-2RA/CD25 at RNA level in these cells (Fig. 2c), the level of CD25 protein does not show a decline in HIV-infected cells at 48 hpi (Extended Data Fig. 2d,e), while the activation of the quiescence programme is clearly detectable at this timepoint (see below). Further, cells showing the transcriptional programme of quiescence could efficiently respond to IL-2 addition (Extended Data Fig. 2f). Thus, acutely HIV-infected cells showed an unexpected pattern of metabolic and transcriptional shutdown and based on downregulation of MTOR, probably translational repression.

**Extended Data Fig. 3:**
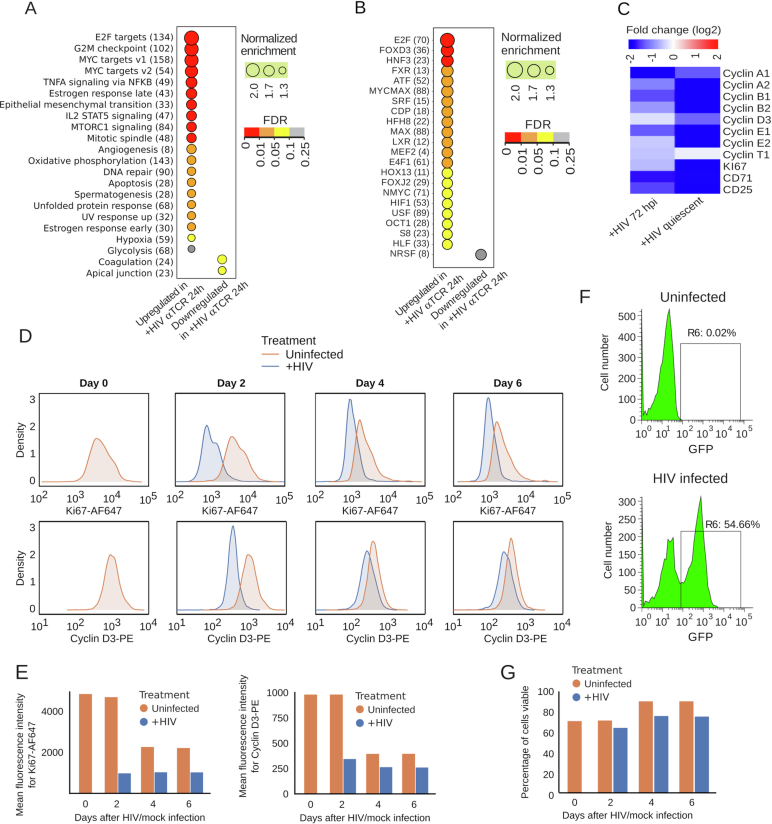
HIV infection results in the downregulation of proliferative pathways. **a., b**. Pathway analysis (**a**) and transcription factor activity analysis (**b**) in +HIV αTCR 24 h cells indicate that many of the pathways upregulated during reactivation from quiescence correspond to those downregulated in +HIV 72 hpi cells. Numbers between parentheses indicate the number of genes in each gene list that drive the enrichment phenotype (see Methods). **c**. Most cyclin genes show a weak downregulation in +HIV 72 hpi cells and a much stronger one upon entry into full quiescence ( + HIV quiescent cells). Proliferation markers Ki67, CD71 and CD25 are included to indicate the extent of inhibition of proliferation. **d**. Relative to uninfected cells, unpurified HIV infected cells show decreased expression of cyclin D3 and Ki67, indicated by flow cytometry analysis performed at 2, 4 and 6 days post-infection. **e**. Mean fluorescence intensity of AF647 anti-Ki67 and PE anti-cyclin D3 signals detectable in uninfected and HIV infected cells over the course of 6 days. **f**. Following exposure of primary CD4 + T cells to HIV reporter virus, over half of the population becomes productively infected based on flow cytometry measurement of the level of expression of the GFP reporter gene. The values shown correspond to 2 days post infection. **g**. Viability of unpurified HIV infected cells relative to uninfected cells at 2, 4 and 6 days post-infection assessed using flow cytometry and Fixable Viability Dye eFluor™ 450. Source data

In contrast, upon addition of quiescence-inducing cytokines to the +HIV cells (Fig. 1a), entry into a fully quiescent state is accompanied by relatively mild changes in key cellular proliferative and metabolic pathways (Figs. 1b and 3a, compare lanes labelled +HIV quiescent and +vector quiescent, also see Extended Data Fig. 3a,b). Consistent with pathway analysis patterns, the number of genes differentially expressed during transition of +HIV 72-hpi cells to full quiescence is small relative to the magnitude of change during entry of +vector 72-hpi cells into quiescence (Fig. 1b). As the fully quiescent +HIV and +vector cells are largely identical in terms of their gene expression pattern as shown by the very small number of genes (<500) differentially expressed between the two (Fig. 1b, also see Fig. 2b), these results indicate that after 72 h of HIV infection, the cells have already undergone a large fraction of transcriptomic changes needed to achieve the fully quiescent state.

**Extended Data Fig. 4:**
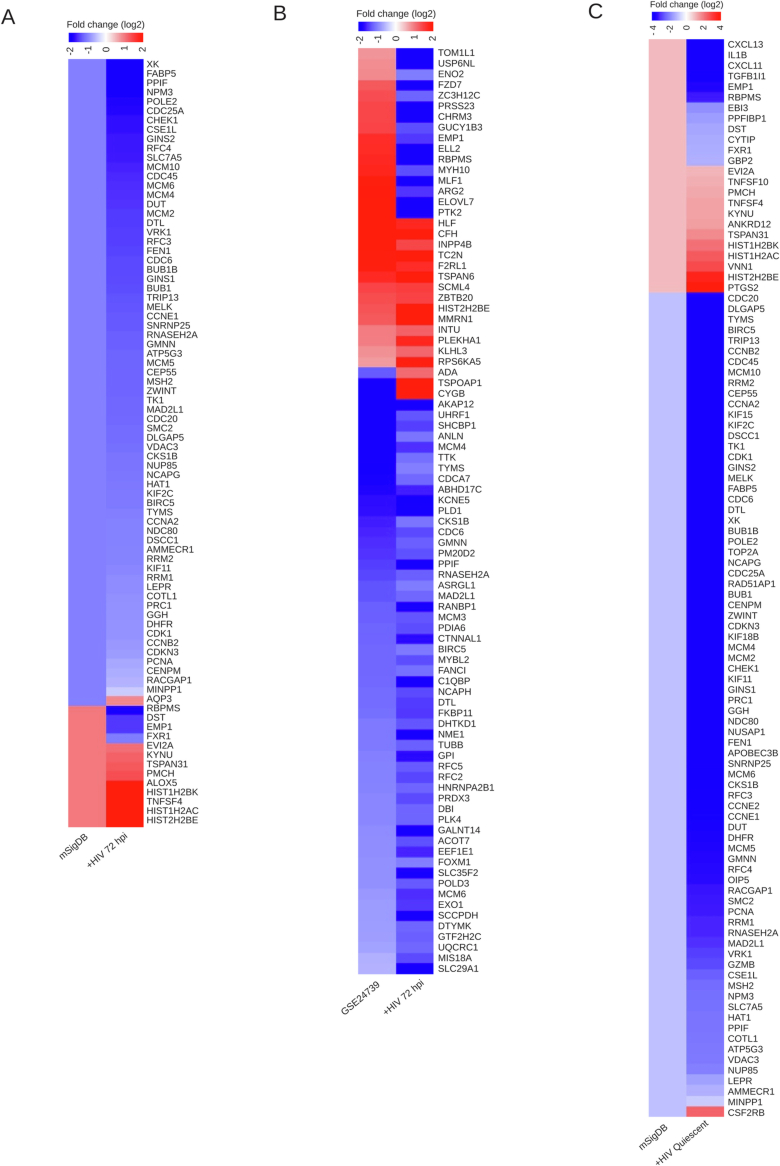
HIV infection leads to the induction of the transcriptomic pattern of quiescence. **a**. HIV infection leads to changes in gene expression that is characteristics of quiescent cells. The column to the left reflects the expected pattern of change in genes that constitute the molecular signature of quiescence. **b**. The pattern of change in gene expression after HIV infection closely resembles that following entry into quiescence in hematopoietic stem cells (lane labeled GSE24739, see Methods). **c**. Pattern of gene expression change after entry of HIV infected cells into full quiescence closely resembles that observed during quiescence in other cell types, as reflected in the quiescence signature pathways in mSigDB. Genes expected to be up or downregulated in quiescence are shown in the lane marked mSigDB with pink and light blue color, respectively. Source data

In summary, transcriptomic results, along with confirmatory western blots (Fig. 2d), indicate that HIV infection led to increased expression of the key quiescence markers KLF2, TOB1 and CDKN2B, along with the downregulation of proliferation markers such as CD25/IL-2RA, CD71/TFRC and MYC (Fig. 2c,d, see also Extended Data Fig. 3c). Validating these unexpected results, a flow cytometry study of markers of proliferation cyclin D3 and Ki67 showed a correlated decrease in protein level in unpurified, minimally disturbed HIV-infected cells compared to uninfected cells starting as early as 48 hours post infection (Fig. 3b and Extended Data Fig. 3d–f). Similarly, analysis of the growth rate of +vector 72-hpi and +HIV 72-hpi cells through calculation of growth curves and CellTrace proliferation assays showed a clear reduction in cellular growth starting at 2 days after HIV infection which reflected the ratio of infected cells in the population, while both groups showed similar viability (Fig. 3c,d and Extended Data Fig. 3f,g). As can be seen in Extended Data Fig. 4a–c, similar to the case with +vector quiescent cells (Extended Data Fig. 1d), the gene expression pattern of +HIV 72-hpi cells closely reflected the transcriptomic signature of the quiescent state.

### HIV infection induces a block to MYC signalling, a key proliferative factor in CD4+ T cells

**Extended Data Fig. 5:**
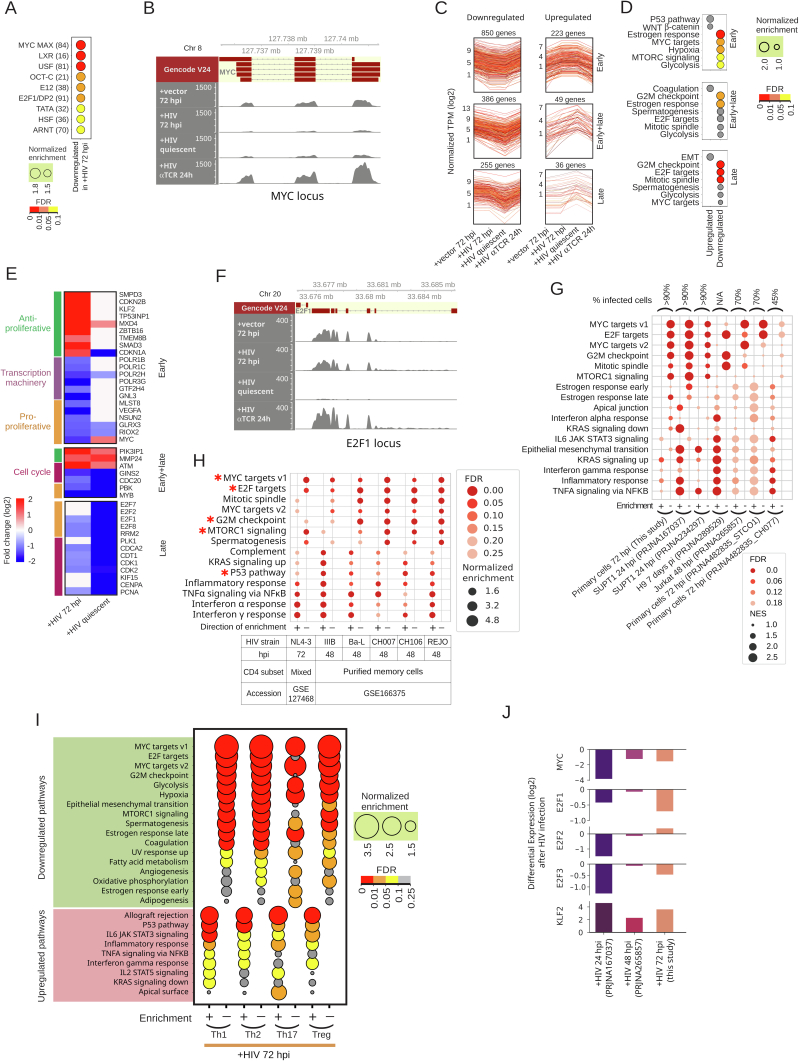
The HIV-induced quiescence is triggered by a wide range of HIV strains and involves a marked downregulation of MYC and its downstream proliferative pathways. **a**. The activity of a number of proliferation-inducing transcription factors, especially MYC, is strongly reduced in +HIV 72 hpi cells. Numbers between parentheses indicate the number of genes in each pathway that drive the enrichment phenotype (see Methods). **b**. Reads mapping to the genomic locus of *MYC* in Th17 cells during different stages in HIV and T cell life cycle. **c**. Temporal pattern of the gene expression changes occurring during the life cycle of HIV infected CD4 + T cells. The number of genes falling into early, late, and early+late categories is written above each box. Genes showing an overall pattern of down- and upregulation are shown in the left and right boxes, respectively. The expression values are graphed as log2 transformed transcript per million (TPM) values, with each line representing a gene. The line graphs indicate the level of expression of each gene in the four cellular states shown at the bottom of each column of line graphs. **d**. Pathway analysis results on genes in each category in panel **c** point to key roles for the p53 pathway and MYC signaling during early timepoints after HIV infection. **e**. Heatmap of the top differentially expressed genes in each category shown in panels **c** and **d**. The color bars to the left show the functional classification of each group of genes. **f**. Reads mapping to the genomic locus of *E2F1* in Th17 cells during different stages in HIV and T cell life cycle. **g**. Pathway analysis using Hallmark gene lists on seven independently performed high throughput transcriptomic studies of CD4 + T cells in early time points after HIV infection point to MYC signaling as the most strongly downregulated pathway after HIV infection. The percentage of infected cells in the sequenced population of each dataset is indicated on top. N/A: not available. Cells used and time point of infection along with project accession numbers are shown at the bottom. The two primary HIV isolates studied in PRJNA482835 (STCO1 and CH077) are graphed separately. **h**. Primary purified CD4+ memory cells infected with five different HIV strains *ex vivo* show the downregulation of MYC, E2F and MTORC signaling along with elevated p53 activity, similar to results obtained using QUECEL models. Asterisks mark pathways differentially enriched concordantly in nearly all shown studies. CD4+ memory cells from three different donors were used in these studies. A similar analysis on a mixed CD4+ population using the NL4-3 HIV strain is also shown. **i**. Pathway analysis results indicate that in all four main subclasses of polarized effector T cells, HIV infection leads to the induction of the signature of entry into quiescence. Plus and minus signs at the bottom indicate positive and negative enrichment of pathways, respectively. **j**. Across multiple high throughput transcriptomic studies, HIV infection leads to strong and reproducible reduction and increase in the expression of MYC and KLF2, respectively. Milder downregulation of E2F family members is observed in some but not all studies. Please note that the Y axis is scaled differently for each of the stacked panels. Source data

We defined the enrichment pattern of transcription factor binding sites near the promoters of genes differentially expressed after HIV infection, which pointed to a strongly significant reduction in expression of genes with binding sites for the two major proliferative transcription factors in CD4+ T cells, MYC and E2F (Fig. 3e and Extended Data Fig. 5a). The temporal pattern of gene expression changes in response to HIV infection and during entry into full quiescence indicated that several key proliferative regulatory pathways and genes, most prominently MYC target genes and MYC itself, show a strong and rapid downregulation predominantly in early timepoints (within 72 h) after HIV infection (Extended Data Fig. 5b–e). A second group of regulatory pathways and genes, such as cyclins and E2F family genes and pathways, are predominantly downregulated in later timepoints (days 4–14 after HIV infection), including during entry into full quiescence after the addition of quiescence-inducing cytokines (Extended Data Fig. 5c–f). Among the upregulated pathways, the p53 pathway and its downstream signalling pathway of Wnt/beta catenin^39,40^ were among the ‘early’ responding group (Extended Data Fig. 5d, see also Fig. 3a). Furthermore, several pro-quiescence genes, including KLF2, CDKN2B, CDKN1A, SMPD3 and SMAD3, were also predominantly or exclusively induced in early timepoints after HIV infection (Extended Data Fig. 5e). Altogether, these data suggest that the majority of the upstream signalling events of the quiescence programme were already set in motion within 72 h post HIV infection, with the gene expression changes at the later timepoints corresponding to the downstream results.

Although the impact of HIV infection on the induction of the transcriptional programme of quiescence has been previously overlooked, individual aspects of this phenotype have been noted in literature including reduced expression of genes associated with activated state in CD4+ T cells^39,41^, suppression of transcription, RNA processing^42^, translation^43^, metabolism, proliferation-related pathways and cellular growth rate^44,45^. To determine the reproducibility and generality of our findings, we used multiple independently performed RNA-seq studies involving early HIV infection timepoints from public databases (Supplementary Table 1) in which high levels of infection were achieved using different strains of replication-competent HIV from LAI and NL4-3 clones^39,41,43,46,47^ and primary HIV isolates^42,44^, which were used to infect diverse CD4+ T cell lines and primary CD4 cells (Supplementary Table 1). Our reanalysis of these additional RNA-seq studies showed changes in critical cellular pathways nearly identical to those in our study. These included an upregulation of the p53 pathway and a downregulation of critical proliferation pathways, most prominently MYC and E2F signalling and cell cycle-related pathways (Extended Data Fig. 5g). We also analysed purified primary CD4+ memory cells infected with six different strains of HIV, all of which showed induction of the HIV-induced quiescence phenotype (Extended Data Fig. 5h).

Finally, we asked whether different polarized CD4+ T cells show differential transcriptomic patterns after HIV infection. While all tested polarized cells showed a strong downregulation of MYC signalling, E2F signalling was not a strong player in Th17 polarized cells (Extended Data Fig. 5i). Interestingly, it has been shown that MYC is a strong regulator of E2F signalling^48,49^. The much smaller magnitude of change in the expression level of the E2F family members that positively regulate T cell proliferation (E2F1, 2 and 3)^50,51^ compared with the level of MYC downregulation following HIV infection (Extended Data Fig. 5b,f,g) suggests that the changes in E2F signalling may be secondary to the reduced level of MYC transcriptional activity.

**Extended Data Fig. 6:**
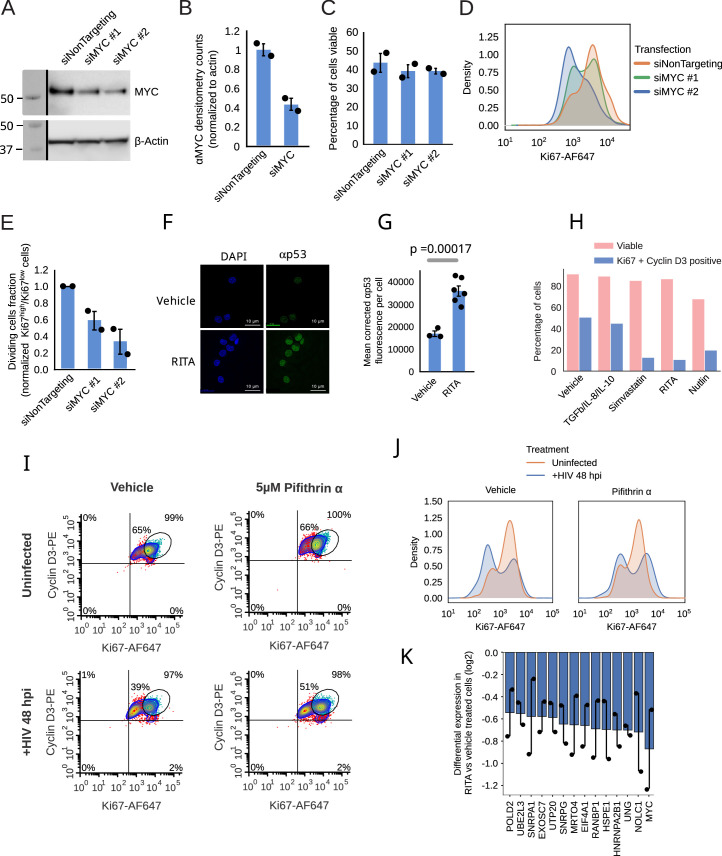
Knockdown of MYC and blocking the p53 pathway activity result in increased and decreased entry into quiescence after HIV infection, respectively. **a**. Immunoblotting analysis indicates that transfection with MYC targeting siRNAs (siMYC) results in decreased MYC protein abundance. Resting memory CD4 + T cells were transfected with nontargeting control or MYC siRNA, then activated via the TCR at 24 hours and harvested at 72 hours post-transfection. Molecular weight markers are shown on the left in kilodaltons. **b**. Densitometry analysis of MYC immunoblotting results. Bars represent the average expression level +/- standard deviation in two knockdown populations from a healthy donor, each normalized to the corresponding expression level of β-Actin. **c**. Viability of control and MYC knockdown cells were assessed using Fixable Viability Dye eFluor™ 450 and flow cytometry performed on the day of cell harvest. Values shown represent mean values of two replicate studies performed independently using cells from a healthy donor, +/- standard deviation. **d**. Histogram representation of the results in Fig. 3g shows the strong reduction in activation-induced expression of cyclin D3 and Ki67 in cells transfected with MYC siRNAs prior to TCR agonist treatment. Immunofluorescent staining and flow cytometry were performed 48 hours after activation. **e**. At 48 hours post-activation, MYC knockdown cells display reduced Ki67 expression when analyzed using flow cytometry compared to non-targeting siRNAs in cells obtained from two independent healthy donors. Values shown represent mean values +/- standard deviation. **f**. The p53 agonist RITA induces nuclear accumulation of p53 in primary CD4 + T cells, demonstrated by confocal fluorescent microscopy images taken at 8 hours post-treatment. Cells are stained with monoclonal p53 antibody (green) and DAPI (blue). **g**. Quantification of anti-p53 antibody fluorescence detected within the nuclear regions of cells obtained from a healthy donor which were imaged at 8 hours post-treatment with vehicle or RITA. Values shown represent the mean signal intensity from cells shown in **f** + /- SEM. Signal intensity difference between DMSO- and RITA-treated cells were assessed using a two-tailed Welch’s t-test, yielding *t*(9) = 7.29, Cohen’s *d* = 3.93, and a 95% confidence interval for the mean difference of [12,811.08, 25,128.77]. **h**. The percentage of viable cells and those double positive for Ki67 and Cyclin D3 are shown for proliferating cells treated with vehicle, TGF-β/IL-8/IL-10 quiescence inducing cocktail, the KLF2 inducer Simvastatin or the p53 agonists RITA or Nutlin. All but the TGF-β/IL-8/IL-10 treated cells were maintained in proliferation supporting media with high levels of IL-2. The shown results are obtained from a single healthy donor. **i**. Flow cytometry results show that the p53 inhibitor pifithrin-α partially inhibits HIV induced loss of cyclin D3 and Ki67 expression when cells are treated with the inhibitor 24 hours prior to HIV infection. **j**. Within 48 hours of HIV infection, both vehicle and pifithrin-α treated cell populations show decreased expression of Ki67, but the decrease is more extensive in the vehicle treated cells. **k**. MYC and several known targets of MYC involved in transcription, translation and cell cycle regulation are downregulated in CD4 + T cells 24 hours after treatment with the p53 agonist RITA. Values represent the mean ± standard deviation from two bulk RNA-seq samples, each generated from cells of an independent healthy donor. Source data

To determine whether downregulation of MYC alone could recapitulate the observed HIV-induced changes in gene expression, we electroporated MYC-targeting and non-targeting control short interfering (si)RNAs into proliferative, uninfected primary CD4+ T cells to knock down MYC (Extended Data Fig. 6a–c). Reduced MYC levels resulted in downregulation of multiple proliferative pathways and a gene expression pattern that very closely mimicked that observed following HIV infection (Fig. 3f). Flow cytometry studies confirmed the loss of proliferative markers in the absence of changes in viability level, similar to what was observed after HIV infection (Fig. 3g,h and Extended Data Fig. 6a,c–e). Thus, the downregulation of MYC was sufficient to recapitulate the gene expression pattern observed after HIV infection.

### Early activation of KLF2 and p53 signalling pathways downregulates MYC and initiates the programme of quiescence

**Fig. 4:**
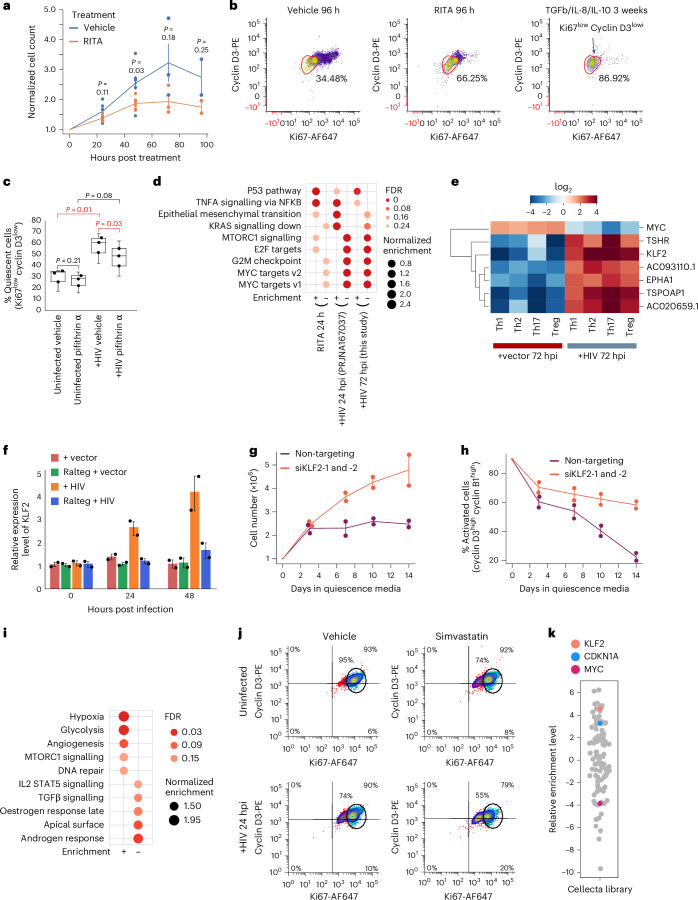
HIV-induced activation of p53 pathway and KLF2 leads to MYC downregulation and loss of proliferation markers. **a**, RIT-induced p53 activation leads to slower proliferation in CD4+ T cells from 2 healthy donors (with 2–3 independent replicates per donor) incubated in proliferation media. The statistical values were based on two-tailed *t*-tests. For the 48 h timepoint, *t*-statistic was *t*(5) = −2.71, 95% confidence interval (CI): [−1.326, −0.037] and Cohen’s *d* = 1.11 (large effect). Here and in panels **f**–**h**, values shown are the mean ± s.d. of biological replicates. **b**, Flow cytometry analysis indicates a strong downregulation of proliferation markers in cells treated with the p53 agonist RITA for 96 h, despite the presence of high IL-2 in the media. Cells treated with vehicle for 96 h or with quiescence-inducing cytokines for 3 weeks (left and right panels, respectively) are included as negative and positive controls. **c**, Pre-treatment of CD4+ T cells from 3 healthy donors with p53 inhibitor pifithrin-α partially inhibits HIV-mediated loss of proliferation markers cyclin D3 and Ki67 in primary CD4+ T cells. In the boxplots: the central line, lower and upper edges of the box correspond to the median, first (Q1) and third (Q3) quartiles, respectively, which in this plot are identical to the data points; the whiskers extend to the most extreme data points within 1.5× the interquartile range (IQR). For the comparisons of uninfected versus +HIV vehicle-treated and +HIV vehicle versus pifithrin-treated samples, two-tailed Student’s *t*-test was used, paired by donor, with the *t*-statistics of *t*(2) = 9.03 and *t*(2) = −5.86, Cohen’s *d*_z_= 5.21 and 3.38 (both large), and 95% CIs = [14.70, 41.44] and [2.61, 17.07] percentage points, respectively. **d**, p53 pathway activation in isolation leads to downregulation of MYC targets and some additional proliferative pathways that are also negatively enriched in HIV-infected cells. For this panel and **i**, plus and minus signs at the bottom indicate positive or negative enrichment. **e**, KLF2 is among transcripts showing the strongest negative correlation with MYC in the main effector cell polarized subtypes. **f**, RT–qPCR analysis of KLF2 level in vector or HIV-infected primary human CD4+ T cells from 2 healthy donors (with 3 PCR replicates per donor) in the presence or absence of Raltegravir (labelled here as Ralteg) confirms the induction of KLF2 after HIV infection and its dependence on proviral integration. **g**,**h**, Knockdown of KLF2 prevents entry into quiescence in CD4+ T cells from 2 healthy donors. Proliferating cells were treated with 2 siRNAs against KLF2 (siKLF2) or a non-targeting control siRNA and were incubated in quiescence-inducing media. Cell number (**g**) or the fraction of cells positive for both cyclins D3 and B1 (**h**) was measured at indicated timepoints. **i**, KLF2 knockdown results in upregulation of pathways associated with proliferation, including mTORC1 signalling and HIF-1α-regulated genes (hypoxia pathway). **j**, Pre-treatment with Simvastatin, a pharmaceutical drug shown to upregulate KLF2, enhances HIV-induced loss of proliferation markers in primary CD4+ T cells. The circular gate marks the location of proliferating cells, with cells inside and outside this gate shown as blue and red dots, respectively. **k**, Unbiased shRNA screen results point to *KLF2* as a gene critical for maintenance of cellular quiescence and proviral latency. Similarly, the negative enrichment of MYC points to its critical role in the maintenance of the cellular proliferative state and/or proviral transcriptional activity. shRNA screen hits are shown as circles, with the *Y* axis indicating relative enrichment. A p53-induced negative regulator of cellular proliferation, CDKN1A/p21, is also enriched. Source data

To define the mechanism of MYC downregulation after HIV infection, we next investigated the potential contribution of HIV-induced early activation of the p53 pathway, a known negative regulator of MYC^52^. Treating proliferating primary human CD4+ T cells with two known p53 agonists, RITA and nutlin^53,54^, led to p53 induction (Extended Data Fig. 6f–h) along with a concomitant slowdown in cellular proliferation and pronounced downregulation of proliferation markers within 24 h (Fig. 4a,b and Extended Data Fig. 6h) with no or minor changes to viability (Extended Data Fig. 6h). Conversely, pre-treatment of cells with pifithrin, a known p53 antagonist, before HIV infection resulted in a significant reduction in the fraction of cells showing a quiescent phenotype (Cyclin D3−, Ki67−) compared to vehicle-treated cells (Fig. 4c and Extended Data Fig. 6i,j). RNA-seq study of the RITA-treated cells pointed to the downregulation of MYC and several MYC target genes (Fig. 4d and Extended Data Fig. 6k), albeit at a lower magnitude compared with what was observed following HIV infection, pointing to the involvement of additional factors in induction of the observed HIV-induced transcriptomic signature of quiescence.

**Extended Data Fig. 7:**
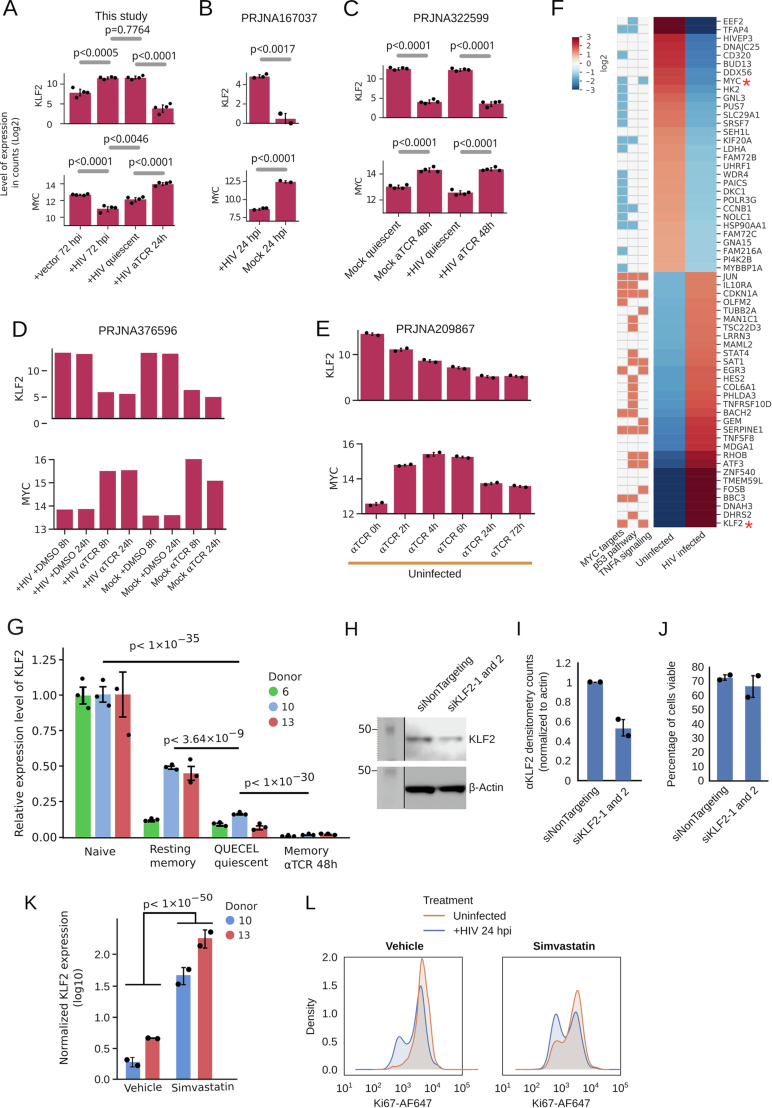
KLF2 plays a key role in entry into quiescence. **a**. KLF2 and MYC show negatively correlated changes in expression during the different stages of HIV and T cell life cycle. The values shown are the average expression of KLF2 or MYC in four independent biological replicates (4 differently polarized cells) per stage of HIV life cycle +/- SEM as error bars. The values originate from a bulk RNA-seq study on cells from a healthy donor. For this experiment and panels **b** and **c**, p-values were calculated using two-tailed paired Student’s T test. **b-e**. Expression of MYC and KLF2 show negative correlation in several independently performed high throughput sequencing studies, including HIV infection in SUPT1 cells (**b**), two primary ex-vivo CD4 + T cell HIV latency model studies (**c**, **d**) and a primary CD4 + T cell activation time course study in the absence of HIV (**e**). Shown values in **b** reflect average values from three biological replicates +/-SD, while those in **c** and **e** correspond to average values from four and two healthy donors, respectively, +/-SD. **f**. Top 30 genes showing the highest level of differential expression in each direction among all datasets studied in Extended Data Fig. 5g include *KLF2* as the most strongly upregulated gene and *MYC* as one of the most downregulated genes (marked by asterisks). The annotation matrix to the left indicates that many of the shown genes are downstream of MYC, p53 and TNF signaling pathways, the top three enriched pathways among those similarly regulated after HIV infection in all datasets studied in Extended Data Fig. 5g. **g**. Ex-vivo polarized Th17 cells treated with the quiescence inducing cytokine cocktail (see Fig. 1a) for 12 days as part of the QUECEL model (QUECEL quiescent) express KLF2 at a level significantly greater than recently *ex vivo*-activated memory cells purified from donors, but less than donor-purified naïve CD4 + T cells and closest to the level observed in donor-purified memory cells, indicating that the KLF2 level in QUECEL models is not outside the physiological range. Data shown reflects average values +/- SD of three independent replicates (two PCR replicates performed with different primer sets, and a third technical replicate made on a different date) performed in cells derived from three independent healthy donors. A linear mixed-effects model was used to account for within-donor correlation by including donor as a random intercept. Log₁₀-transformed KLF2 expression was modeled as a function of experimental condition, with QUECEL quiescent cells specified as the reference group. Compared to QUECEL quiescent cells, memory T cells showed the smallest and least significant difference (β = 0.481, 95% CI [0.321, 0.641], z = 5.90, p = 3.64×10⁻^9^). In contrast, naive T cells exhibited a much larger increase in KLF2 expression (β = 1.011, 95% CI [0.851, 1.171], z = 12.40, p < 1×10⁻^35^), and restimulated memory T cells showed markedly reduced expression (β = −0.943, 95% CI [ − 1.103, −0.783], z = −11.57, p < 1×10⁻^30^). These results indicate that among all groups, memory cells were the most similar to QUECEL quiescent cells in KLF2 expression. **h**. Immunoblotting analysis indicates that transfection with KLF2 targeting siRNAs (siKLF2-1 and siKLF2-2) results in decreased KLF2 protein abundance. Th17 cells were transfected with nontargeting control or the two KLF2-targeting siRNAs one week after TCR activation, then incubated in the quiescence inducing cytokine cocktail and harvested 48 hours later. Molecular weight markers are shown on the left in kilodaltons. **i**. Densitometry analysis of KLF2 immunoblotting results. Bars represent the mean ± SD of expression levels for each siRNA condition, calculated from two independent healthy donors. The conditions include a non-targeting siRNA and a mixture of two distinct KLF2-targeting siRNAs. For each condition, values from the two donors were first normalized to the corresponding β-actin expression level and then averaged. **j**. Viability of control and KLF2 knockdown cells, corresponding to those in panel **i**, assessed using Fixable Viability Dye eFluor™ 450 and flow cytometry performed on the day of cell harvest. **k**. KLF2 expression is upregulated by more than 20-fold in Th17 cells treated with 10 µM Simvastatin for 24 hours. Values shown correspond to average of two independent technical replicates (two sets of PCR primers) per donor and two biological replicates (two independent healthy donors) +/- SD. A linear mixed-effects model was fit to log10-transformed KLF2 expression levels, with fixed effects for treatment, primer sets used, and their interaction, and a random intercept for donor. Simvastatin treatment significantly increased KLF2 expression (β = 1.36 log10 units, 95% CI [1.18, 1.53], corresponding to an estimated ~22.7-fold increase. Wald z-tests were used for inference. **l**. Flow cytometry results represented as a histogram demonstrate that at 24 hours post-infection with HIV, cells pre-treated with Simvastatin show strong loss of Ki67 expression, while cells pre-treated with vehicle are still in the early phase of the quiescence transition. Source data

To find potential candidate genes that may play such a role, we identified the HIV-induced genes that showed a negative correlation with MYC level (Fig. 4e). Interestingly, KLF2, a known potent negative regulator of MYC and a key inducer of cellular quiescence in CD4+ T cells, was among the genes showing the strongest negative correlation with MYC in multiple studies both in the presence and absence of HIV (Fig. 4e and Extended Data Fig. 7a–e). Among the ~100 protein-coding genes that were consistently up or downregulated after HIV infection in datasets shown in Extended Data Fig. 5g, the most upregulated gene was *KLF2* (Extended Data Fig. 7f). In contrast, *MYC* was among the genes consistently showing a strong downregulation after HIV infection (Extended Data Fig. 7f).

Timecourse studies in primary human CD4+ T cells following infection with the HIV reporter virus indicated that the level of KLF2 was significantly upregulated at 24 hpi and showed further increase at 48 hpi (Fig. 4f), reaching levels comparable to those in resting memory cells (Extended Data Fig. 7g). Importantly, this increase was not a passive side effect of cellular stress but depended on HIV proviral integration into the genome, as raltegravir addition blocked the rise in KLF2 levels (Fig. 4f). KLF2 knockdown prevented entry into quiescence, even when the knockdown cells were cultured in quiescence-inducing media, without affecting viability (Fig. 4g,h and Extended Data Fig. 7h–j). Pathway analysis pointed to induction of pathways that are downregulated during entry into quiescence, including glycolysis, MTORC signalling and HIF1a downstream genes (hypoxia pathway)(Fig. 4i). On the other hand, treating proliferating primary human CD4+ T cells with simvastatin, a known inducer of KLF2 (refs. ^55–57^), strongly reduced the expression of proliferative markers without affecting cell viability (Fig. 4j, and Extended Data Figs. 6h and 7k,l), consistent with exit from the proliferating state.

To complement the above studies, we also performed an unbiased short hairpin RNA (shRNA) screen against 15,000 cellular protein-coding genes to identify those involved in positive or negative regulation of HIV transcriptional activity^58^. Since proviral activation is strongly associated with the T cell activated state^11^, loss of function of factors involved in inhibiting T cell activation will yield positive hits in our screen. Interestingly, ~40 genes among those consistently up or downregulated following HIV infection (Extended Data Fig. 7f) were enriched in the shRNA screen, with *KLF2* and *MYC* among the most positively and negatively enriched genes in this list, respectively (Fig. 4k). *CDKN1A/p21*, a known p53 effector gene and a potent inducer of cell cycle arrest, was also among the positively enriched genes (Fig. 4k). Interestingly, an independently performed shRNA screen study identified KLF2 as a key factor in maintaining HIV latency^59^. Together, these data not only indicate that KLF2 and MYC are among the most differentially regulated genes and pathways after HIV infection, but also that their loss of function is sufficient to induce and prevent HIV latency reversal, respectively, probably through regulation of the balance between the activated and quiescent state in CD4+ T cells.

### Single-cell (sc)RNA-seq demonstrates that activation of KLF2 and p53 pathways is associated with proviral transcriptional shutdown

**Fig. 5:**
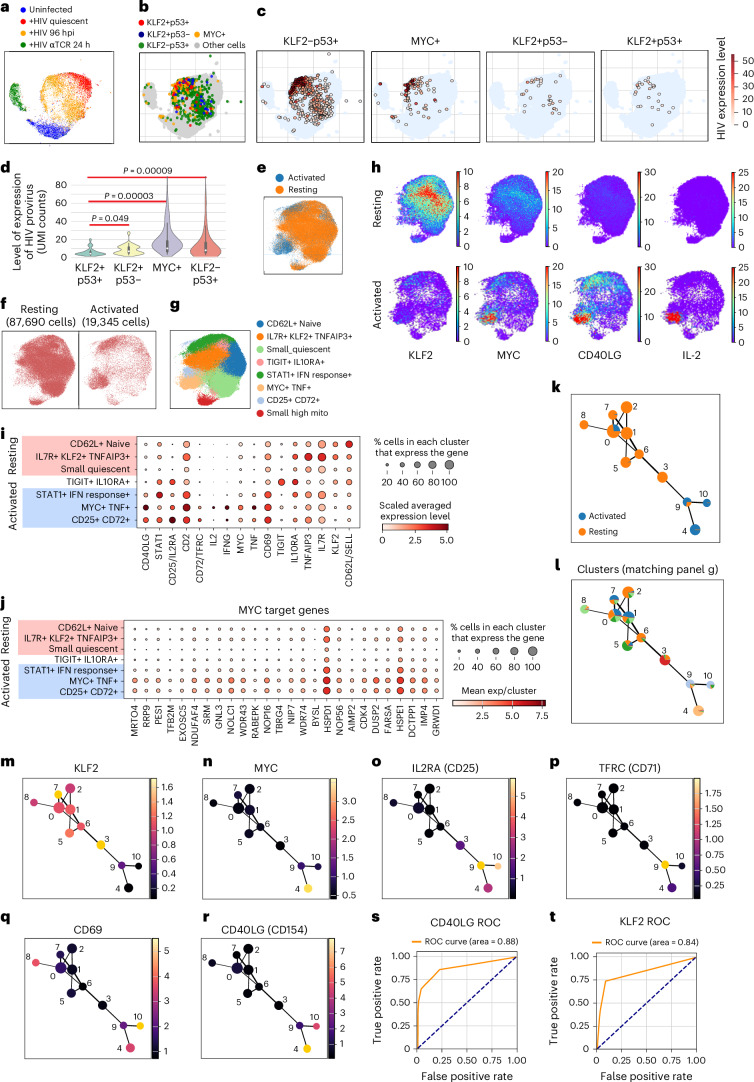
Transcriptional silencing of the HIV proviral genome by concomitant activation of p53 signalling and KLF2, a key transcription factor associated with the quiescence/resting state, after HIV infection. **a**, Uniform manifold approximation and projection (UMAP) of scRNA-seq data derived from a healthy donor with 2 biological replicates in addition to technical replicates shows HIV+ 96-hpi cells clustering between uninfected proliferating cells and HIV+ quiescent cells. **b**, Distribution of +HIV 96-hpi cells expressing MYC, KLF2, p53 pathway genes, or both p53 pathway genes and KLF2. **c**, Level of expression of HIV provirus in cells expressing p53 pathway genes, MYC, KLF2 or p53 pathway genes and KLF2 in combination. **d**, Proviral transcriptional shutdown is associated with dual activation of p53 and KLF2 signalling in ex vivo HIV-infected CD4+ T cells. In each violin plot, the central box, white dot and whiskers indicate the interquartile range (25th–75th percentiles), median and 1.5× IQR. Two-tailed Mann–Whitney *U*-test was used for the comparisons of KLF2+p53+ vs KLF2+53−, MYC+ and KLF2−p53+, yielding *U* statistics of 293.0, 667.5 and 4,541.5, respectively. As the primary hypothesis tested was that the KLF2+p53+ group differed from all three comparator groups, no multiple-comparison correction was required (intersection–union framework). **e**, UMAP of single-cell RNA-seq data from 6 HIV+ donor CD4+ T cells. Colours show the separation of resting and ex vivo activated cells. **f**, Side-by-side UMAPs show the distribution of resting and ex vivo activated cells. **g**, Clustering study showing the presence of different resting and activated states. **h**, Expression pattern of key quiescence and activation markers on UMAPs identifies 3 clusters which largely consist of activated cells. The color bar indicates log1p-transformed gene expression values. **i**, Dotplot identifies clusters representing activated, resting and intermediate states. **j**, Dotplot showing the strong induction of MYC target genes in the 2 most activated and inflamed clusters. **k**, Trajectory analysis indicates the separation of the cells into untreated (largely KLF2+ resting cells before ex vivo reactivation) and ex vivo reactivated groups, with the majority of the reactivated group in nodes 4, 9 and 10. **l**, The two MYC+ clusters in **j** constitute over 80% of cells in nodes 4, 9 and 10 which correspond to the endpoint of the trajectory, highlighting the strong association of MYC with the reactivated state. Node colors correspond to the cluster identities defined in **g**, and the pie chart proportions within each node represent the relative contributions of each cluster. **m**,**n**, Expression pattern of KLF2 and MYC indicate their association with the resting and activated state, respectively. Colour bars to the right of each plot indicate the averaged expression level per node. **o**–**r**, Expression patterns of multiple markers of the activated state map to nodes 4, 9 and 10. **s**,**t**, Calculations of the AUC of ROC plots indicate that the expression patterns of CD40LG and KLF2 are excellent predictors of the activated and quiescent states, respectively.

**Extended Data Fig. 8:**
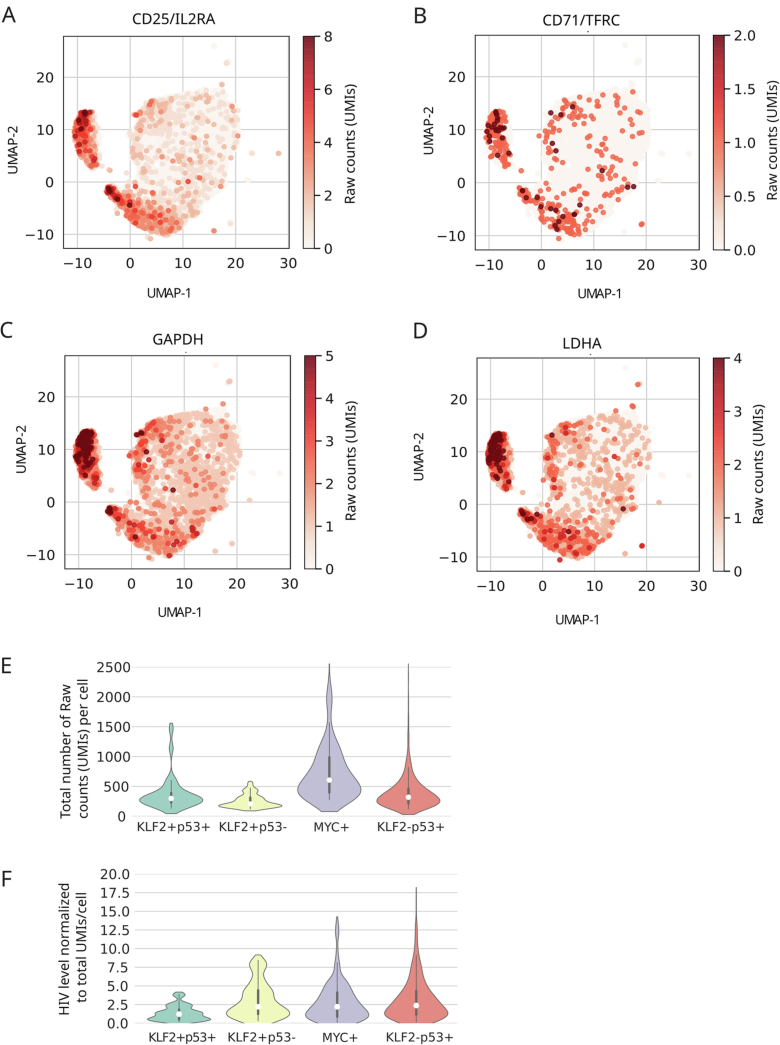
Single-cell RNA-seq points to the exclusion of markers of T cell activated state from HIV-infected cells. **a**-**d**. UMAP projection of single-cell RNA-seq data shows that cells with high levels of cellular activation markers CD25 and CD71 (**a, b**) and metabolic markers GAPDH and LDHA (**c, d**) are found predominantly among uninfected and reactivated HIV+ cell clusters, consistent with the lower proliferation and metabolic activity in +HIV 96 hpi and quiescent cells. **e**. The total number of UMIs (unique molecular identifiers) per cell, which is an indirect measure of cell size and total number of cellular transcripts, is shown for cells in each category. A smaller value is associated with quiescence in T cells. In this panel and **f**, the shown data is obtained from single-cell RNA-seq on a healthy donor, and in each violin plot, the central box indicates the interquartile range (25th–75th percentile), the white dot within the box marks the median, and the whiskers extend to 1.5× the interquartile range. **f**. Dual activation of p53 and KLF2 signaling leads to the lowest proviral transcriptional activity even when the HIV counts (UMIs) in each cell are normalized to total counts (UMIs) per cell.

We also performed scRNA-seq analysis on primary CD4+ T cells from a healthy donor with two biological replicates in addition to technical replicates. We included samples before infection, 96 hpi with our HIV reporter virus, following 14 days of incubation of the infected cells in the quiescence-inducing media, and 24 h after reactivation of the quiescent cells through TCR stimulation (+HIV αTCR 24 h). As with bulk RNA-seq results (Fig. 2a), dimensionality reduction studies indicated that +HIV 96-hpi cells that were maintained in proliferative media were distinct from the identically maintained uninfected cells and occupied a position between the uninfected and quiescent cells (Fig. 5a). Multiple proliferation and metabolic activation markers were negatively enriched in the +HIV quiescent and 96-hpi cells compared to proliferative uninfected and +HIV αTCR 24-h cells (Extended Data Fig. 8a–d). Analysis of the HIV expression level in +HIV 96-hpi cells indicated that those with a signature of MYC expression had the highest HIV expression level (MYC+ cells, Fig. 5b–d). These cells also had the highest unique molecular identifier (UMI) counts per cell, consistent with a more activated phenotype (Extended Data Fig. 8e). Cells expressing p53 activation markers in the absence of KLF2 expression had a lower number of cellular RNAs consistent with a more quiescent phenotype, but only a modestly reduced HIV expression level, indicating that induction of p53 signalling per se is not sufficient to transcriptionally silence the provirus (KLF2–p53+ cells; Fig. 5c,d and Extended Data Fig. 8e). In contrast, KLF2-expressing cells, even in the absence of p53 signalling, had a much lower number of RNAs per cell and limited the proviral transcription to a basal level (KLF2+p53– cells; Fig. 5c,d and Extended Data Fig. 8e). Importantly, cells in which both KLF2 and the p53 signalling were activated showed a strong reduction of HIV proviral transcriptional activity, with an average of >75% reduction in proviral transcription compared to MYC+ cells (KLF2+p53+ cells; Fig. 5c,d and Extended Data Fig. 8e). This strongly reduced proviral transcription level in KLF2+p53+ cells was observed both before and after normalization of proviral expression level to total cellular counts (Extended Data Fig. 8f). Thus, the post-infection concurrent activation of the KLF2 and p53 pathways, in addition to reprogramming of cells for entry into quiescence, also sharply reduced the expression of proviral genes, setting the stage for proviral latency and formation of the latent reservoir.

**Extended Data Fig. 9:**
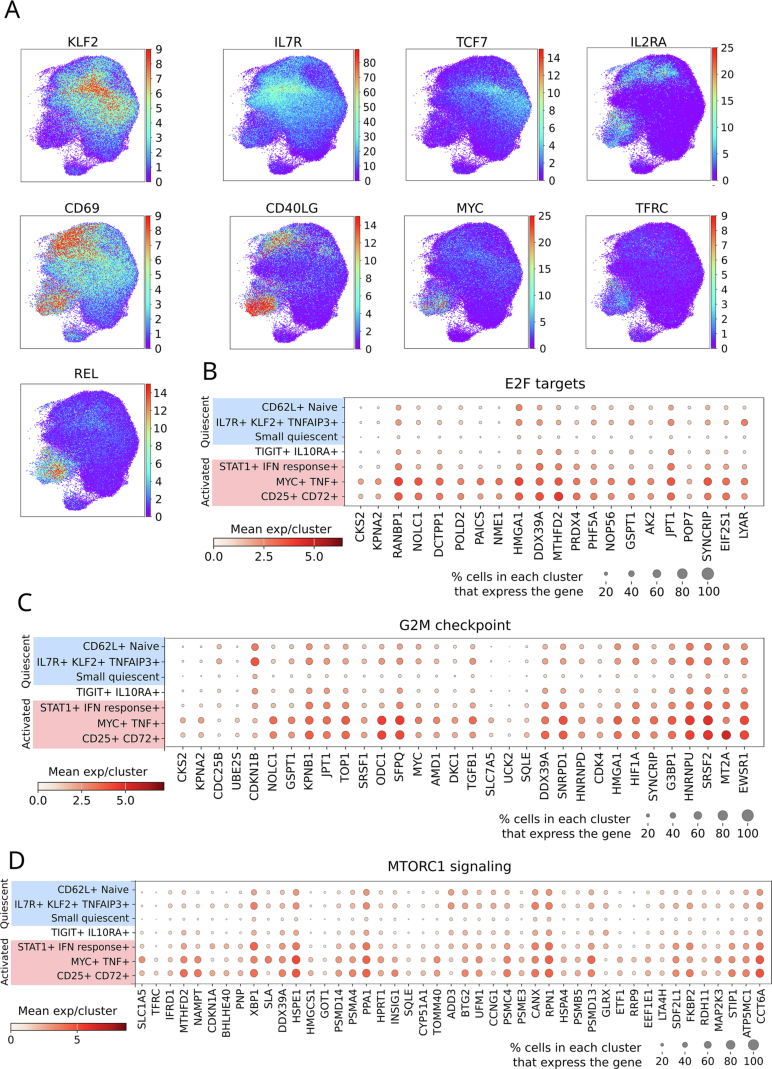
KLF2 and MYC are associated with resting and activated state in CD4 + T cells from people living with HIV. **a**. UMAPs show the expression of KLF2, IL7R and TCF7 in resting CD4 + T cells from people with HIV, while the activated clusters show the expression of IL2RA (CD25), CD40LG, MYC, TFRC and REL. **b-d**. Dot plots show the expression pattern of key pathways associated with CD4 + T cell activated state. The identity of clusters corresponding to each row is shown on the left. The size of circles correspond to the fraction of cells expressing the genes in each pathway. The intensity of color corresponds to the mean expression level of the genes involved in each pathway, aggregated for each cluster.

**Extended Data Fig. 10:**
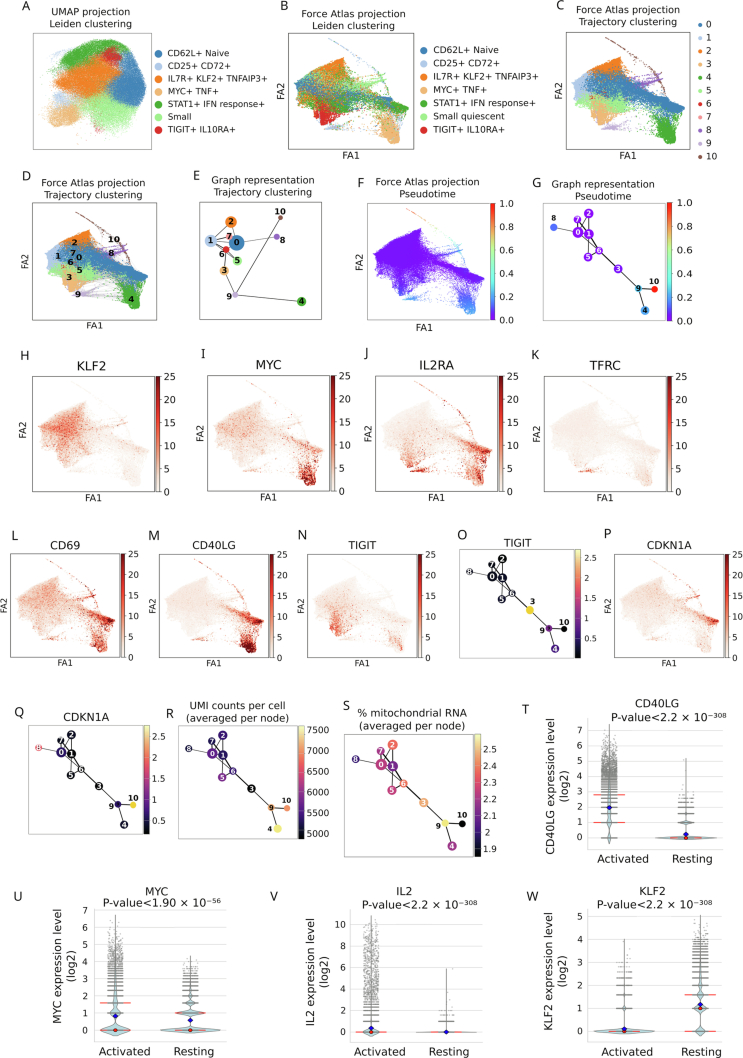
Trajectory analysis reveals the association of KLF2 and MYC with the quiescent and activated state, respectively, in HIV+ donor CD4 + T cells. **a**. UMAP projection with the coloring scheme showing the clusters corresponding to the functional clustering marking different classes of activated and resting cells, which are derived using the leiden algorithm. **b**. Force Atlas projection of the same clusters (leiden clustering). **c** and **d**. Force Atlas projection with the clustering used for the trajectory analysis. The thin lines connecting cells (shown as dots) represent the deduced trajectories. The new clusters are marked by numbers, with the largest cluster (the one with the highest number of cells in it) being cluster zero and the smallest, cluster 10. Colors show the cells belonging to each cluster. **e**. Simplified representation of the clusters shown in panel **d**, as a graph plot. The numbers next to each node correspond to the number associated with the clusters in panel **d**. Node size correlates with the number of cells in the cluster represented by each node, with larger nodes corresponding to clusters with more cells. The nodes are colored to match the coloring scheme in **c** and **d** panels. **f**. Force Atlas projection, with the pseudotime calculations shown as a color transition. The color bar on the right shows the colors corresponding to the start of the trajectory (dark blue) and its end (red). **g**. The pseudotime superimposed on the linearized graph plot. **h**-**m**. Force Atlas projections showing the expression of markers of resting and activated CD4 + T cells on the trajectory. The color bar on the right of each panel shows the expression level. For matched graph plots, see Fig. 5m-r. **n**-**q**. Matched Force Atlas and graph plots depicting the expression patterns of TIGIT and CDKN1A. **r**. Graph plot with UMI counts per cell represented by color. As expected, nodes corresponding to activated cells (nodes 4, 9 and 10) have higher UMI counts, which generally correlates with the number of transcripts found in each cell. **s**. Neither of the clusters on the trajectory contain a high percentage of mitochondrial RNA, which is associated with dead/dying cells. **t**-**w**. Violin plots showing the expression level of key markers of resting and activated state in resting and activated CD4 + T cells from six HIV+ donors. The numbers shown are based on raw UMI counts. The use of raw, non-normalized counts is needed as transition between the resting and activated state results in changes in the size of the transcriptome. Violin plots display the distribution of expression values for each group; the outline depicts the probability density of the data at different values, red horizontal lines indicate the first and third quartiles, red circles denote the medians, and blue diamonds indicate the means. Comparisons between activated and resting cells for each transcript were performed using a two-sided Mann–Whitney U test with no adjustment for multiple comparisons. For KLF2, U = 135,959,975.5, rank-biserial correlation = 0.84, 95% CI [0.83, 0.85]; for CD40LG, U = 737,704,123.5, r = 0.79, 95% CI [0.78, 0.80]; for MYC, U = 447,742,560.5, r = 0.15, 95% CI [0.14, 0.16]; and for IL2, U = 461,422,717.5, r = 0.69, 95% CI [0.68, 0.70]. Source data

Finally, we used primary CD4+ T cells from the Sabes project^60^, which includes cells from 6 HIV-positive donors both at the viraemic state and following a year of cART-mediated suppression. We analysed scRNA-seq data obtained at basal state and following ex vivo antigen-mediated reactivation in the presence of 10 μM enfuvirtide for 9 h, which led to the induction of HIV transcription^60^. At basal state, cells showed a high KLF2 level and a gene expression pattern consistent with the quiescence state, including a low MYC level (Fig. 5e–h and Extended Data Fig. 9a). The level of MYC and other markers of T cell activation, including IL-2 and CD40LG, showed a dramatic increase after reactivation, along with a strong reduction in KLF2 levels (Fig. 5h–j and Extended Data Fig. 9a–d). Trajectory analysis indicated the presence of multiple quiescent populations corresponding to quiescent naive and memory cells representing the starting point of the pseudotime (Fig. 5k,l and Extended Data Fig. 10a–g). Following reactivation, three distinct populations of cells at various activated states were detected, representing the downstream stages of the pseudotime, with the MYC^high^ CD40LG^high^ KLF2^low^ cells constituting the main population of reactivated cells (Fig. 5l–r and Extended Data Fig. 10h–s). To determine the extent of association of KLF2 expression with the resting/quiescent state in CD4+ T cells, we trained a logistic regression model using the Sabes RNA-seq dataset, with CD40LG, a strong marker of the activated state in CD4+ cells used as control. As expected, logistic regression models proved CD40LG to be a strong predictor of the activated state (Extended Data Fig. 10t–v and Supplementary Table 2). KLF2 similarly performed strongly as a predictor of the quiescent/resting state, with a coefficient of +3.3 (confidence interval: 3.24–3.42). Accuracy, precision and recall values of 0.83, 0.89 and 0.74, respectively, proved that KLF2 is an accurate and sensitive marker of quiescent/resting cells (Extended Data Fig. 10w and Supplementary Table 2). Consistently, receiver operating characteristic (ROC) curves showed area under the curves (AUC) of 0.84 and 0.88 for KLF2 and CD40LG, respectively, proving both to be excellent predictors of the resting and activated state, respectively (Fig. 5s,t).

**Fig. 6:**
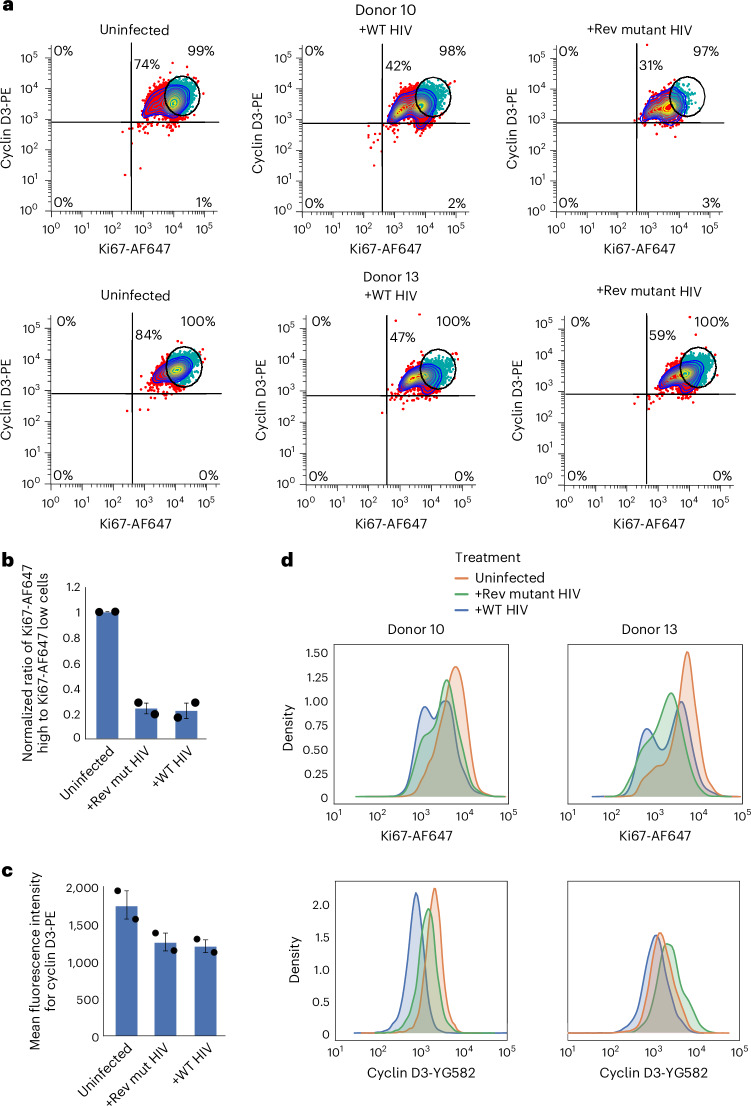
A loss-of-function mutation within the *Rev* gene does not abolish the quiescence-inducing capacity of HIV. **a**, Ex vivo infection of Th17 polarized primary CD4+ T cells from 2 healthy donors with a Rev mutant HIV construct results in loss of cyclin D3 and Ki67 expression within 48 h. This indicates that *Tat* and/or *Nef*, which are the only genes still expressed by this construct, are critical for the induction of the HIV-induced quiescence programme. **b**, The Rev mutant HIV construct induces the same level of drop in Ki67 as the parental construct with a functional Rev in 48-hpi ex vivo-infected CD4+ T cell populations from 2 healthy donors. The ratio of cells with high versus low levels of Ki67 as shown by flow cytometry is plotted on the *Y* axis. In this panel and in **c**, the values shown are the mean ± s.d. of biological replicates. **c**, Mean fluorescence intensity of PE anti-cyclin D3 signal detectable in uninfected cells and cells infected with wild-type or mutant Rev HIV constructs at 48 hpi from 2 healthy donors indicates a similar level of loss of proliferative state. **d**, Flow cytometry results represented as a histogram show the bimodal pattern of expression of Ki67 at 48 hpi in ex vivo-infected cells from 2 healthy donors, consistent with the activation of the HIV-induced quiescence programme in the Rev mutant HIV construct. Source data

We have not yet defined which HIV gene(s) are involved in the induction of the HIV-mediated quiescence programme. However, Tat appears to contribute to the phenotype. We used a virus that was unable to express the *Rev* gene due to mutation of the initiator methionine to threonine in our HIV construct. This mutation effectively prevents the expression of the HIV proteins except for Tat and Nef. Infection of human primary CD4+ cells with this mutant construct and one containing wild-type *Rev* gene led to a comparable induction of the quiescence programme (Fig. 6a–d), indicating that Tat may contribute to the induction of the HIV-induced quiescence programme. This is consistent with reports that soluble Tat has anti-proliferative effects due to the induction of IL-10 (refs. ^61,62^). However, the indirect effects of Tat due to the induction of apoptotic pathways could also contribute to these phenotypes^63^.

## Discussion

Our data reveal a previously unknown facet of the formation of the latent reservoir, in which the expression of proviral proteins, probably Tat, shortly after infection with HIV and proviral integration results in the activation of a multipronged quiescence mechanism. This process, which is mediated through activation of the pro-quiescence p53 signalling cascade and KLF2 among other factors, leads to suppression of the expression of MYC, which is known to play a pivotal role in maintaining the activated state of T cells^35,64^. The resulting downregulation of key proliferative and metabolic pathways, in turn, lead to cellular quiescence and proviral transcriptional shutdown at least in a subset of the infected cells.

It has been shown that shortly after HIV infection, induction of the IFN response and cellular stress result in p53 activation^65,66^. In addition to its pro-quiescent function, p53 can directly restrict HIV replication and proviral gene expression through multiple mechanisms^67–70^, which may contribute to the observed proviral transcriptional shutdown. While the induction of p53 leads to apoptosis in a fraction of HIV-infected cells^71^, a steep rise in the expression of pro-quiescence, pro-survival factor KLF2 after HIV infection leads to survival and induction of quiescence in CD4+ T cells^20,21,25^. In contrast to p53, the mechanism of induction of KLF2 after HIV infection is not known, and the absence of KLF2 upregulation after treatment of primary CD4+ T cells with IFNα did not support the possibility that similar to p53, upregulation of KLF2 is induced by the innate immune response (Supplementary Fig. 2). FOXO1, a critical factor in homeostasis of CD4+ T cells, has been shown to play a role in induction of KLF2 (ref. ^72^). It is plausible that cellular stress and/or DNA damage response following viral integration leads to activation of FOXO1 (refs. ^73,74^), which in turn induces the expression of KLF2. Our observation that proviral integration is necessary for KLF2 induction after HIV infection is consistent with this possibility. However, additional signals from the expressed proviral genes, probably Tat, are needed for the induction of KLF2 and the quiescence phenotype, as cells transfected with an empty lentiviral vector did not show a rise in KLF2 level compared to the control.

Accumulating evidence from our work and others^7,9–11^ documents the close association of entry into quiescence with proviral latency. While many aspects of the initiation and progression of this HIV-induced quiescence programme remain to be studied, our observation of pro-quiescence reprogramming of CD4+ T cells shortly after HIV infection suggests that the formation of a population of latently infected cells is an inherent outcome of HIV infection, pointing to the inexorability of the formation of the latent reservoir.

## Methods

The studies described in this manuscript, including the use of human peripheral blood mononuclear cells (PBMCs) and HIV viral constructs, comply with all relevant biosafety and ethics regulations and have been approved by Case Western Reserve University’s Institutional Review Board (IRB No. 01-98-55). For a description of reagents used in this work, please see Supplementary Table 3.

### Primary cell culture

Peripheral blood mononuclear cells from HIV-negative Caucasian male donors were purchased from Allcells (ordering code LP, CR, MNC, 100 M). Naive and memory CD4+ T cells were isolated using EasySep Human Naïve CD4+ T Cell Isolation kit II and EasySep Human Memory CD4+ Cell Enrichment kit, respectively, according to manufacturer’s instructions. Cells were resuspended at 1 × 10^6^ cells per ml in primary culture media (RPMI with Normocin, 10% fetal bovine serum and 25 mM HEPES). Immediately after isolation, cells were treated with Dynabeads Human T-Activator CD3/CD28 at a 1:1 bead-to-cell ratio and 60 IU ml^−1^ IL-2. Cells were maintained at 37 °C and 5% CO_2_. Dynabeads were removed using a magnet after 24 h. To polarize naïve CD4+ T cells into the four major effector T cell subsets Th1, Th2, Th17 and Treg cells, purified naive CD4 T cells were resuspended in 10 ml RPMI medium, stimulated with 10 μg ml^−1^ concanavalin A (ConA, EMD Millipore) and exposed to subset-specific cytokines. After 72 h at 37 °C, additional fresh medium, ConA, polarization cocktail cytokines and 120 IU ml^−1^ of IL-2 was added. After an additional 6 days, the cells were washed and transferred into primary cell RPMI medium containing growth cytokines of IL-23 (50 ng ml^−1^) and IL-2 (60 IU ml^−1^) for Th17 cells, while for Th1, Th2 and Treg cells, only IL-2 was added^11^. During subsequent expansion, cell populations were diluted back to 1 × 10^6^ cells per ml in primary culture media and supplemented with 60 IU ml^−1^ IL-2 every 2 days.

### Cell lines

Jurkat cells were purchased from ATCC (TIB-152) and maintained at 37 °C and 5% CO_2_ in the described primary culture medium. Authentication documents are available through the vendor upon request.

### Reporter virus

A single-round VSV-pseudotyped HIV reporter virus included the *tat*, *rev*, *env*, *vpu* and *nef* genes and a CD8-EGFP fusion protein that permits the use of magnetic beads for a gentle, minimally disturbing purification of infected cells (Fig. 1a). As with many membrane-bound proteins, the CD8-EGFP fusion protein has a long half-life, thus permitting the purification of infected cells even after entering latency. This pseudotyped reporter virus cannot form syncytia, so it does not lead to the strong cytopathic effects observed with full-length, replicative competent HIV.

### Reporter virus infection

Ten million polarized CD4+ T cells at a concentration of 5 × 10^6^ cells per ml were mixed with high-titre single-round VSV-pseudotyped HIV reporter virus and centrifuged at 1,800 × *g* for 1.5 h to promote viral fusion. Cells were incubated overnight in the existing media, then diluted back to 1 × 10^6^ cells per ml in primary culture media and supplemented with 60 IU ml^−1^ IL-2. At 72 h post reactivation, the infected cells were purified with minimal disturbance using magnetic beads. The purified cells were >95% positive for the viral Nef protein and GFP^11^.

### Quiescence and reactivation of CD4+ T cells

To induce quiescence, on day 7 post infection, the purified, HIV-infected (+HIV) or identically maintained uninfected or vector-infected cells were switched to media containing a defined cocktail of quiescence-inducing cytokines including 10 ng ml^−1^ TGFβ, 50 ng ml^−1^ IL-8 and 10 ng ml^−1^ IL-10 (ref. ^11^). Every 4 days, these cytokines were re-added along with 15 IU ml^−1^ IL-2 to maintain cell viability. To reactivate the quiescent cells, they were stimulated through the TCR using CD3/CD28 beads for 24 h, as described above.

Flow cytometry assays confirmed entry into quiescence by measuring the progressive reduction in proliferation markers and sharply reduced proliferation rate^11^. Once quiescent, the level of HIV expression is reduced to almost undetectable levels (1%), indicative of HIV latency. Upon stimulation through the TCR by exposure to α-CD3/α-CD28 monoclonal antibodies, the fraction of HIV-expressing cells increases dramatically (>80%), indicative of exit from quiescence and proviral latency.

### RT–qPCR assays

For RT–qPCR-based assays, total cellular RNA was collected using TRIzol reagent and preparation of complementary (c)DNA was performed with PrimeScript RT Reagent kit as described, using both oligo(dT) and random hexamers^75^. The resulting cDNA was used in qPCR reactions with iQ SYBR Green Supermix on an Eppendorf Mastercycler Realplex2 system and analysed as described^75^. For studies with Raltegravir, either vehicle or Raltegravir was added to the proliferating primary CD4+ T cells at a final concentration of 5 μM 48 h before infection with the vector or HIV reporter viral preparations, followed by collection of cellular RNA at the indicated timepoints after infection.

The primers used for detection of MYC and KLF2 in these studies were:

KLF2 FWD 1: CAAACGCACCGCCACTCACAC

KLF2 RVS 1: AGCCGTCCCAGTTGCAGTGGTA

KLF2 FWD 482: CTACACCAAGAGTTCGCATCTG

KLF2 RVS 482: CCGTGTGCTTTCGGTAGTG

MYC FWD 1: GGACCCGCTTCTCTGAAAGGCT

MYC RVS 1: TAACGTTGAGGGGCATCGTCGC

MYC FWD 2: CCACCACCAGCAGCGACTCT

MYC RVS 2: CCTTTTGCCAGGAGCCTGCCTC

### Bulk RNA-seq library preparation

Three replicate bulk RNA-seq experiments were performed over 2 years. Briefly, poly(A)+ RNA (replicates 1 and 2) or total cellular RNA (replicate 3) were collected from a million viable Th1, Th2, Th17 and Treg cells 72 h after infection with the HIV construct shown in Fig. 1a containing a GFP-CD8a fusion reporter gene. For total cellular RNA preparations, ribosomal RNAs and other abundant housekeeping RNAs were removed, followed by fragmentation, cDNA synthesis and adaptor ligation^11^. High-throughput sequencing was performed on an Illumina HiSeq2000 instrument.

### scRNA-seq library preparation

Aliquots of ~600,000 cells were taken for scRNA-seq analysis at each of the following timepoints: before the infection with HIV reporter viruses (Uninfected), 96 h after infection (+HIV 96 hpi), 14 days after the initial quiescence-inducing cytokine treatment (+HIV quiescent) and 24 h after reactivation using anti-CD3/CD28 beads (+HIV αTCR 24 h). The Drop-seq protocol was performed according to the McCarroll laboratory guidebook^76^. Barcoded mRNA capture beads were used at a concentration of 120,000 beads per ml in lysis buffer prepared as described. Cells were used at 100 cells per µl in PBS containing 10% bovine serum albumin. Flow rates were set to 2,000 µl h^−1^ for cells and beads, and 7,500 µl h^−1^ for oil. The beads were washed with 6× SSC and the 5× RT buffer supplied with Maxima H minus reverse transcriptase. Reverse transcription, treatment with Exonuclease I, PCR amplification and tagmentation were performed strictly as described in the Drop-seq guidebook^76^ using the Maxima H minus reverse transcriptase, Exonuclease I and Nextera XT DNA Library Preparation kit. Next-generation sequencing of the DNA libraries was performed using the Illumina HiSeq platform at Medgenome.

### Defining the quiescence-related gene signatures

To identify genes that change in a concordant manner during quiescence in different cell types capable of entry into a quiescent state, we selected quiescence-related pathways in the mSigDB that were derived from non-cancerous cells, along with a related published study (accession number: GSE24739) on quiescence in primary haematopoietic stem cells^36^. The resulting gene list was used as a signature of gene expression patterns in quiescent cells and was compared to the datasets analysed in this study.

### shRNA screen

The shRNA screen using the Cellecta shRNA library (HGW-M1-P2) was performed according to manufacturer’s instructions^58^. Briefly, Jurkat T cells harbouring a latent GFP reporter-containing HIV provirus (E4 cells) were infected with the shRNA library, and GFP+ cells were screened for constitutive activation of the latent provirus through a sequencing-based approach. This approach identified genes that, when knocked down, resulted in T cell reactivation and/or HIV proviral activation. A reverse screen was performed in which cells were stimulated with TCR agonists, resulting in enhanced proliferation of Jurkat cells and reactivation of the latent provirus. Cells that did not reactivate the provirus were selected to identify the genes for which knockdown resulted in a block to T cell proliferation and/or proviral reactivation. The combined results of the dual screen were matched against the genes showing concordant expression among all early-timepoint HIV infection RNA-seq datasets, and the resulting list of genes was evaluated for positive or negative enrichment.

### Flow cytometry analysis

Cells were washed with PBS and treated with Fixable Viability Dye eFluor 450 for 15 min, then washed with PBS again. Cells were fixed in 4% formaldehyde and permeabilized with Perm/wash buffer. To detect cyclin D3 and Ki67, cells were incubated with 3 µg ml^−1^ AF647 mouse anti-Ki67 (Biolegend, 350509, RRID:AB_10900810) and 3 µg ml^−1^ PE mouse anti-cyclin D3 (Biolegend, 684903, RRID:AB_2686979) for 15 min. Perm/wash buffer was used to wash the cells twice after incubations. Fluorescence signals were measured using a BD LSRFortessa flow cytometer. Antibody authentication document is available from the vendor upon request.

### Analysis of cellular proliferation

To assess the proliferation of RITA- and vehicle-treated cell populations, viable cell count was measured at the same time each day using trypan blue stain and a Countess II FL automated cell counter. To compare the proliferation rate of HIV-infected and uninfected populations, the CellTrace Yellow proliferation assay was used. Freshly isolated naïve CD4+ T cells were resuspended in PBS at a concentration of 1 × 10^6^ cells per ml with 4 µM CellTrace Yellow dye (ThermoFisher, C34567). The solution was incubated at 37 °C with constant agitation for 20 min, then diluted 3-fold with RPMI to quench the staining reaction. After 5 min, cells were resuspended in primary culture media, stimulated through the TCR, polarized and infected as described above.

### RITA, Nutlin, pifithrin α and simvastatin treatments

To upregulate p53, cells were treated with the small-molecule MDM2 inhibitors RITA and Nutlin. RITA was used at a concentration of 10 µM for Th17 cells (Fig. 4d and Extended Data Fig. 6f–h) or 1.5 µM for unpolarized CD4+ T cells more susceptible to RITA toxicity (Fig. 4a,b). The RITA concentrations used were empirically determined so that cell viability will not be significantly compromised. Nutlin was used at 10 µM concentration. To inhibit p53 before HIV infection, cells were incubated with 5 µM pifithrin α for 24 h. To upregulate KLF2, cells were treated with 10 µM simvastatin for 24 h before HIV infection or data collection in uninfected populations. As a control for each drug treatment, a parallel population was treated with an equivalent volume of dimethylsulfoxide (DMSO), the vehicle used for drug delivery.

### Knockdown studies on MYC and KLF2

Dicer-substrate short interfering RNAs (dsiRNAs) targeting MYC, KLF2 or non-targeting sequences were transfected into primary CD4+ T cells using either electroporation or chemical transfection. For electroporation, one million cells were washed with PBS and resuspended in 60 µl MaxCyte electroporation buffer. DsiRNAs were added at 3 µM concentration. For MYC and KLF2, two different dsiRNA constructs were used as a mixture. A TEX615-labelled non-targeting dsiRNA was applied at the same concentration as a transfection efficiency control. Electroporation was performed using the MaxCyte STX scalable transfection system. Cells were incubated at 37 °C for 15 min in the existing buffer, then resuspended at 1 × 10^6^ cells per ml in primary culture media with 60 IU ml^−1^ IL-2 or the quiescence-inducing cytokine cocktail described above. Fluorescent signal from transfection efficiency control dsiRNA was detected using a BD LSRFortessa flow cytometer. For chemical transfection, 12 pmol of each dsiRNA was combined with 400 µl Opti-MEM and subsequently mixed with 15 µl INTERFERin reagent. The solution was incubated at room temperature for 15 min, then delivered to populations of 3–5 million cells in 2 ml primary culture media. Cells were incubated at 37 °C for 4–6 h, then diluted with fresh primary culture media to a concentration of 1 × 10^6^ cells per ml and supplemented with 60 IU ml^−1^ IL-2 or quiescence-inducing cytokine cocktail.

Information on dsiRNAs used in this study (sourced from IDT) is as follows:

**Table:** 

Scrambled negative control dsiRNA (non-targeting)	IDT, 51-01-19-08
TEX 615 transfection control dsiRNA	IDT, 51-01-20-21
MYC-targeting dsiRNA #1	5’-AUCAUUGAGCCAAAUCUUAAAAAAA
	5’-UUUUUUUAAGAUUUGGCUCAAUGAUAU
MYC -targeting dsiRNA #2	5’-CGACGAGACCUUCAUCAAAAACATC
	5’-GAUGUUUUUGAUGAAGGUCUCGUCGUC
KLF2-targeting dsiRNA #1	5’-GUGCAAUAAUUUAAGUGG
	5’-GAAGAUGCCACUUAAAUU
KLF2-targeting dsiRNA #2	5’-CGAGGCUUGUGAUGCCU
	5’-UUCUCACAAGGCAUCACA

### Immunofluorescence cytochemistry assays

For immunofluorescence detection of p53 following RITA treatment, 200,000 cells were fixed in 4% formaldehyde at 8 h post treatment, then permeabilized with Perm/wash buffer. To detect p53, cells were incubated with rabbit anti-p53 primary antibody (Abcam, ab32389, RRID:AB_776981) at 7 µg ml^−1^, then AF647 goat anti-rabbit secondary antibody (Abcam, ab150079, RRID:AB_2722623) at 7 µg ml^−1^ for 15 min each. DAPI was also applied at 1 µM concentration for 5 min. Perm/wash buffer was used to wash the cells twice after each incubation. Cells were transferred to 0.17-mm poly-l-lysine-coated coverslips and mounted onto microscope slides with ProLong Diamond Antifade mountant. Images were collected using a DeltaVision deconvolution microscope.

### Analysis of bulk RNA-seq data

An average of ~50 million single-end (replicates 1 and 2) or paired-end reads (replicate 3) were obtained for each sample. RNA-seq reads were quality controlled using Fastqc and trimmed for any leftover adaptor-derived sequences and sequences with a Phred score <30 with Trim Galore, which is a wrapper based on Cutadapt and FastQC. Any reads <40 nucleotides after the trimming were not used in alignment. The pre-processed reads were aligned to the human genome (hg38/GRCh38) with Gencode release 27 as the reference annotations using STAR (v.2.7.2b)^77^, followed by gene-level quantitation using htseq-count^78^. In parallel, the pre-processed reads were pseudoaligned using Kallisto (v.0.43.1)^79^ with 100 rounds of bootstrapping to Gencode release 27 of the human transcriptome to which the sequence of the transfected HIV genome and the deduced HIV spliced transcripts were added. The resulting quantitations were normalized using Sleuth. The two pipelines yielded concordant results. Pairwise differential expression tests were performed using edgeR (QL)^80^, and false discovery rate (FDR) values were calculated for each differential expression value.

### Jensen–Shannon distance (JSD)

JSD is a method for measuring the similarity between two probability distributions. In the context of bulk RNA-seq samples, it can be used to compare the gene expression profiles of different samples and was performed as implemented in edgeR. A JSD value close to 0 indicates that the two RNA-seq samples have very similar gene expression profiles, while a higher JSD value indicates greater dissimilarity between the samples.

### Multidimensional scaling (MDS)

MDS is a statistical technique used to visualize the level of similarity or dissimilarity of data points in a low-dimensional space. It is particularly useful for exploring high-dimensional data, such as gene expression profiles from RNA-seq experiments. In the context of RNA-seq data, MDS can help to visualize the relationships between different samples on the basis of their gene expression profiles and was performed as implemented in edgeR. MDS aims to place each sample in a low-dimensional space (typically 2D or 3D) such that the distances between samples in this space reflect their original dissimilarities as closely as possible. The goal is to preserve the pairwise distances, so similar samples (that is, those with similar gene expression profiles) will be close to each other in the MDS plot, while dissimilar samples will be farther apart.

### Pathway analysis

Protein-coding genes expressed at a minimum abundance of 5 transcripts per million (TPM) were used for pathway analysis, with fold-change values as the ranking parameter while controlling the FDR at 0.05. The gene set enrichment analysis (GSEA) package was used to identify the enriched pathways and promoter elements using collections C2, C3, C5 and Hallmark from the mSigDB. Pathways that showed an FDR *q*-value ≤ 0.25 were considered significantly enriched following the GSEA package guidelines. The number of genes contributing to the enrichment score was calculated using the leading edge output of GSEA (tag multiplied by size).

Publicly available datasets focusing on early timepoints after HIV infection were analysed as described above, and the results were compared to the published manuscript associated with the dataset, when applicable, which showed complete agreement between our analysis and the results reported in the corresponding manuscripts. Datasets that showed a >50% infection rate were included in the study to capture the transcriptomic pattern of the infected, rather than bystander, cells. Genes that showed concordant differential expression in all datasets were selected, and the average of differential expression values was used to identify the top shared differentially expressed genes.

### Analysis of scRNA-seq data

Genomic alignment was performed using kallisto-BUS^81^, followed by analysis using the Scanpy suite of packages^82^. A total of 24,894 cells were used in the final analysis, and at least 200 genes were detected in each cell. This low cut-off value was chosen to ensure that the quiescent cells, which are small and thus have a smaller number of genes represented, were included in the analysis population. To determine the expression level of critical factors involved in the regulation of the quiescence programme, the expression level of HIV provirus was calculated, along with the expression of KLF2, MYC and factors that comprised the signature of p53 and MYC activation. As MYC mRNA has one of the shortest known half-lives for an mRNA, the expression of MYC and its downstream transcriptional targets including MKI67/ki67, TFRC/CD71, CCND3/cyclin D3 and IL-2RA/CD25 were used in conjunction with MYC RNA itself to identify cells showing the signature of MYC activity (MYC+ cells), defined as cells showing the expression of any two of the above factors. For determining the signature of p53 activation in early timepoints after HIV infection, the p53 gene list from the curated Hallmark database of mSigDB was used. Genes included in the ‘negative regulation of p53’ in the GO database were removed from the list. The remaining genes were intersected with the list of genes induced after HIV infection in at least four of the datasets in Supplementary Table 1. The resulting list of genes (*TOB1*, *CDKN1A*, *BBC3*, *S100A10*, *HINT1*, *EEF2*, *LDHB*, *BTG1*, *IP6K2*) was used for identifying cells carrying the signature of p53 activation (p53+ cells), defined as cells showing the expression of any two of the above-listed factors. Analysis of the genes directly involved in the p53-mediated induction of apoptosis indicated that they were almost absent from the p53+ cells. Cells that showed the expression of both MYC and KLF2 signatures (observed in only two cells), or both MYC and p53 signature genes as detailed above, were eliminated from the analysis. Those showing the expression of both KLF2 and p53 signature genes were placed in the KLF2+p53+ category. Studies performed before and after normalization of gene expression values to total cellular counts (UMIs) yielded highly similar results. The results obtained using raw reads, which are more pertinent to the study of proviral quiescence, were used to generate the figures reported in this study.

### Trajectory analysis

Trajectory analysis in RNA-seq data is a method used to understand cellular states’ dynamic processes and progression. It helps to reconstruct the paths that cells follow over time or through differentiation processes. It was performed as implemented in the partition-based graph abstraction (PAGA) algorithm^83^ within the Scanpy suite. PAGA is a graph-based approach providing an abstract representation of scRNA-seq data. It identifies and visualizes the connectivity between clusters of cells, which helps in understanding the transitions and trajectories between different cell states. In the graph constructed by PAGA, nodes represent clusters of cells, and edges represent the connectivity or transition probabilities between these clusters.

Methods: Single-cell RNA-seq data were pre-processed and normalized using Scanpy and clustered using the Louvain algorithm to identify distinct cell states. For the trajectory analyses performed on the Sabes project scRNA-seq data^60^, the cluster labelled ‘Small high mito’, which contained apoptotic/dying cells, was not included as it did not represent a cellular state of interest. The connectivity between clusters was computed, and the graph abstraction was generated using PAGA to represent the connectivity and transitions between cell states. The inferred PAGA graph was used to determine the most likely trajectories between clusters, representing the progression and branching of cellular states. Pseudotime calculation in PAGA was performed after identifying a root cell, calculating shortest paths from the root and assigning pseudotime values to cells on the basis of their distance from the root. This approach provides a robust method for mapping the dynamic progression of cellular states, offering valuable insights into developmental processes and differentiation pathways.

### Logistic regression and predictor analysis

Logistic regression: Univariate logistic regression models were employed to assess the predictive value of *KLF2* for determining the quiescent cellular state. *CD40LG*, a known marker and predictor of the activated state, was used as a control. The expression levels of the two genes were extracted and standardized using the StandardScaler from the scikit-learn library. Scaling was performed independently within each cross-validation fold to prevent data leakage. Activated and resting states were assigned on the basis of whether a sample was subjected to ex vivo activation or left untreated. To address the class imbalance in the original dataset, we implemented a balanced subsampling approach by using the smaller class size as the subsample size for both classes. Cells were randomly selected from the larger class to create a balanced subset. We performed separate logistic regression analyses for each gene (*KLF2* and *CD40LG*, along with *MYC* and *IL-2* as additional controls) to examine its relationship with the resting or activation state. A fixed random seed was set before each sampling or model fitting step to ensure the reproducibility of results. Different seed values were used for different operations to maintain independence. Logistic regression models were fitted using the logit function from the statmodels library to obtain regression coefficients, confidence intervals and *p* values.

Cross-validation: To evaluate the performance and robustness of the logistic regression models, 5-fold stratified cross-validation was employed to obtain robust estimates of model parameters using StratifiedKFold. The dataset was randomly partitioned into five equal subsets, maintaining the balance between activated and quiescent cells in each fold. For each iteration, a logistic regression model was fitted using statsmodels’ Logit function. The model was trained on four-fifths of the data and validated on the remaining fifth. This process was repeated five times, ensuring that each subset served as the test set exactly once. After cross-validation, a final model was fitted to the entire dataset to obtain stable coefficient estimates. Confidence intervals (95%) for coefficients were calculated using the profile likelihood method to assess the statistical significance of each gene’s predictive power. Independence of observations was assumed on the basis of the nature of single-cell data, where each cell represents an independent observation.

### Model evaluation

Performance metrics: For each gene, predicted probabilities from the logistic regression models were used to calculate the ROC curves and the AUC values. The ROC curves were plotted using the roc_curve function from the scikit-learn library, and AUC values were calculated using the auc function. In addition to ROC and AUC, other performance metrics, such as accuracy, precision, recall and F1 score, were computed using scikit-learn functions. These metrics provided a comprehensive evaluation of each gene’s predictive capability.

### ROC and AUC analyses

ROC curves were generated for each gene to visualize the trade-off between sensitivity (true positive rate) and specificity. The ROC curve plots the true positive rate (TPR) against the false positive rate (FPR) at various threshold settings. The AUC was calculated to quantify the overall ability of the gene expression levels to discriminate between ‘resting’ and ‘activated’ cells. An AUC value of 1 indicates perfect discrimination, while 0.5 indicates no discrimination (random guessing).

### Quantification and statistical analysis

All experiments were performed with at least three technical replicates and two or more biological replicates, which yielded similar results. All error bars shown in the figures correspond to either standard deviation or standard error of the mean, as noted in figure legends. Depending on the specific structure of the data, the most suitable statistical analysis was chosen, including paired or unpaired *t*-tests, Mann–Whitney *U*-test and linear mixed-effect models. The two-tailed Mann–Whitney *U*-test, which is non-parametric and does not require a normality assumption, was used to compare gene expression patterns in bulk and scRNA-seq studies. For *t*-test and linear model analyses, the normality of residuals was assumed but not formally tested. No statistical methods were used to predetermine sample sizes, but our sample sizes are similar to those reported in previous publications^84,85^. The samples used were not randomized in our studies, as our analysis method used a paired analysis strategy with donors as the pairing variable. Data collection and analysis were not performed blind to the conditions of the experiments. No samples or data points were excluded from the analyses performed except as noted.

### Reporting summary

Further information on research design is available in the Nature Portfolio Reporting Summary linked to this article.

## Supplementary information

Supplementary Information Supplementary Figs. 1 and 2, including gating strategy figure and Tables 1–3.

Reporting Summary

Supplementary Data 1 Source data for Supplementary Fig. 2.

## Source data

Source Data Fig. 1 Statistical source data.

Source Data Fig. 2 Statistical source data.

Source Data Fig. 2 Unprocessed western blots.

Source Data Fig. 3 Statistical source data.

Source Data Fig. 4 Statistical source data.

Source Data Fig. 6 Statistical source data.

Source Data Extended Data Fig. 1 Statistical source data.

Source Data Extended Data Fig. 2 Statistical source data.

Source Data Extended Data Fig. 3 Statistical source data.

Source Data Extended Data Fig. 4 Statistical source data.

Source Data Extended Data Fig. 5 Statistical source data.

Source Data Extended Data Fig. 6 Statistical source data.

Source Data Extended Data Fig. 6 Unprocessed western blots.

Source Data Extended Data Fig. 7 Statistical source data.

Source Data Extended Data Fig 7 Unprocessed western blots.

Source Data Extended Data Fig. 10 Statistical source data.

## Data Availability

The RNA-seq studies used in this work are available through accession numbers SRP145508 and PRJNA1297811. Publicly available datasets used in this manuscript can be accessed through accession numbers SRP013224 (ref. ^41^), SRP075608 (ref. ^34^), SRP100643, SRP026389, GSE187515 (ref. ^60^), GSE127468 (ref. ^86^), GSE166375 (ref. ^87^), SRP035316 (ref. ^39^), SRP060668 (ref. ^47^), SRP049410 and SRP155217 (ref. ^44^). Source data are provided with this paper.
