# Supplementary material for: HIV infection reprogrammes CD4+ T cells for quiescence and entry into proviral latency
Source: Nat Microbiol. 2025 Sep 26;10(10):2454–71. doi: 10.1038/s41564-025-02128-y (PMC12488490; doi:10.1038/s41564-025-02128-y)
Supplement: Supplementary file 1 — Supplementary Figs. 1 and 2, including gating strategy figure and Tables 1–3. [file 41564_2025_2128_MOESM1_ESM.pdf]

nature microbiology

Supplementary information https://doi.org/10.1038/s41564-025-02128-y

HIV infection reprogrammes CD4+ T cells for quiescence and entry into proviral latency

In the format provided by the authors and unedited

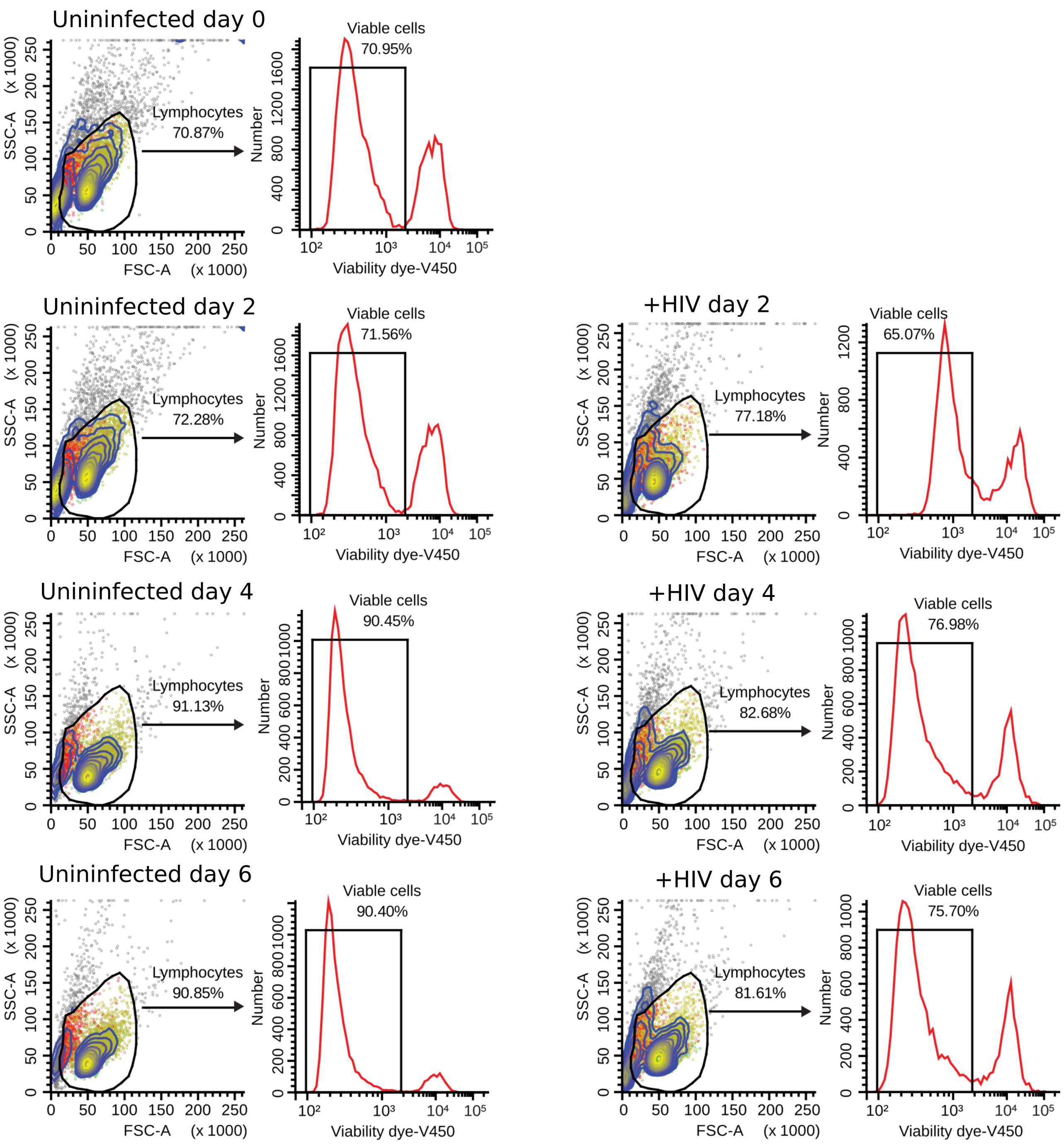

Supplementary Fig. 1. Flow cytometry gating strategy for viable lymphocyte identification. Representative flow cytometry plots showing the sequential gating strategy used for all flow cytometry analyses in this manuscript. Left panel: Forward scatter (FSC-A) vs. side scatter (SSC-A) plot used to identify the lymphocyte population (black contour gate) while excluding most debris, dead cells, doublets, granulocytes, and other non-lymphocytic cell populations that fall outside the lymphocyte scatter profile. Right panel: Histogram of viability dye V450 fluorescence intensity used to exclude any remaining dead cells within the lymphocyte gate, identifying viable cells by low dye uptake (black rectangular gate). This two-step gating strategy was consistently applied across all flow cytometry experiments. Data shown are from Fig. 3B.

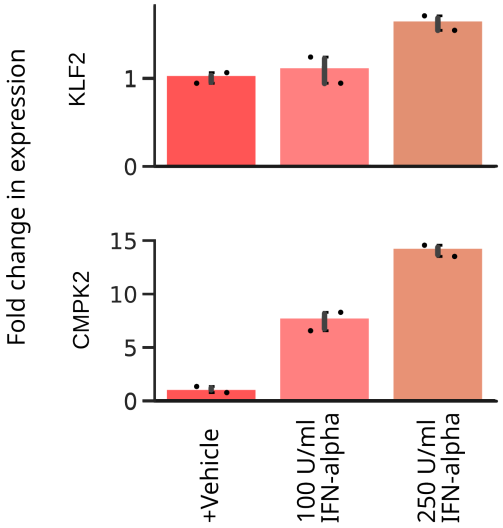

Supplementary Fig. 2. Interferon (IFN) pathway activation does not lead to KLF2 induction. Primary human T cells obtained from a healthy donor were treated with 100 or 250 U/ml of IFNα for 9 hours, followed by harvest and RT-qPCR assay. Two replicate studies were performed on different dates. CMPK2, an IFN responsive gene, was used as control. Shown data represent average of the two replicates +/- standard deviation.

Supplementary Table 1: list of RNA-seq datasets studying the impact of early HIV infection in T cells.

Accession #	Cells used	HIV strain used	Time point after HIV infection	Reported infection rate
PRJNA265857	Jurkat	HIV-1 NL4-3	48 hours	~70%
PRJNA289529	H9	HIV-1 NL4-3	7 days	Not reported
PRJNA234297	SUPT1	HIV-1 LAI	12 and 24 hours	Close to 100%
PRJNA167037	SUPT1	HIV-1 LAI	12 and 24 hours	Close to 100%
PRJNA482835	Primary CD4+ T cells	CH077	72 hours	60-80%
PRJNA482835	Primary CD4+ T cells	STCO1	72 hours	30-60%
This study	Primary CD4+ T cells	HIV-1 NL4-3, single round reporter virus	72 hours	>95%

Supplementary Table 2. Metrics from the logistic regression analysis of association of KLF2 and CD40LG with the quiescent and activated state, respectively. The regression coefficient, including its confidence interval (Conf. Int.) from 2.5 to 97.5% are shown. This value represents the change in the log-odds of a cell being in the resting (for KLF2) or activated state (for CD40LG) for a one-unit increase in gene expression. A positive coefficient means that higher expression of the gene is associated with a higher probability of the cell being in the resting (for KLF2) and activated (for CD40LG) state. The magnitude of the coefficient indicates the strength of this association. Accuracy value provides an overall measure of how well the gene's expression predicts the resting (for KLF2) or activated (for CD40LG) state. Precision value indicates the proportion of true positive predictions (correctly predicted resting/activated cells) among all positive predictions. Recall (also known as Sensitivity) is the proportion of true positive predictions among all actual resting/activated cells. F1 Score is the harmonic mean of precision and recall, with a high F1 score providing a summary metric indicating that the gene is a good predictor of the resting/activation state.

	Gene	Coefficient	Conf. Int. 2.5%	Conf. Int. 97.5%	Accuracy	Precision	Recall	F1 Score	P-value
For predicting resting/quiescent cells:									
	KLF2	3.33	3.24	3.42	0.83	0.89	0.74	0.81	< 0.00001
For predicting activated cells:									
	CD40LG	10.2	9.93	10.48	0.81	0.9	0.69	0.78	< 0.00001
	MYC	0.58	0.54	0.62	0.55	0.61	0.26	0.36	< 0.00001
	IL2	83.2	74.06	92.34	0.55	0.97	0.11	0.2	< 0.00001

Supplementary Table 3. Reagents used in this study

REAGENT or RESOURCE	SOURCE	IDENTIFIER
Antibodies		
AF647 mouse anti-Ki67	Biolegend	Cat#350509, RRID:AB_10900810
PE mouse anti-Cyclin D3	Biolegend	Cat#684903, RRID:AB_2686979
Rabbit anti-p53	Abcam	Cat#ab32389, RRID:AB_776981
AF647 goat anti-rabbit	Abcam	Cat#ab150079, RRID:AB_2722623
Dynabeads™ Human T-Activator CD3/CD28	Thermo Fisher	Cat#11161D
Biological Samples		
Human peripheral blood mononuclear cells	Allcells	N/A
Chemicals, Peptides, and Recombinant Proteins		
IL-2	NIH	Cat#136
TGF-β	PeproTech	Cat#100-21C
IL-8	PeproTech	Cat#200-08M
IL-10	PeproTech	Cat#200-10
TRIzol reagent	ThermoFisher	Cat#15596026
Raltegravir	Millipore Sigma	Cat#CDS023737
Fixable Viability Dye eFluor™ 450	ThermoFisher	Cat#65-0863-14
Perm/Wash™ buffer	BD Biosciences	Cat#554723
CellTrace™ yellow	ThermoFisher	Cat#C34567
RITA	Cayman Chemical	Cat#10006426
Nutlin	Cayman Chemical	Cat#18585
Pifithrin-α	Millipore Sigma	Cat#506132
Simvastatin	Cayman Chemical	Cat#10010344
MaxCyte electroporation buffer	MaxCyte	N/A

INTERFERin®	Polyplus	Cat#101000028
ProLong™ Diamond Antifade Mountant	ThermoFisher	Cat#P36961
Maxima H minus reverse transcriptase	ThermoFisher	Cat#EP0751
Exonuclease I	New England Biolabs	Cat# M0293S
Critical Commercial Assays		
EasySep™ Human Naïve CD4+ T Cell Isolation Kit II	Stem Cell Technologies	Cat#17555
EasySep™ Human Memory CD4+ T Cell Enrichment Kit	Stem Cell Technologies	Cat#19157
PrimeScript RT Reagent Kit	TAKARA Bio	Cat#RR037B
iQ™ SYBR® Green Supermix	Biorad	Cat#1708880
Nextera XT DNA Library Preparation Kit	Illumina	Cat# FC-131-1024
Deposited Data		
T cell polarization protocol	(Dobrowolski et al., 2019)	DOI: 10.1128/mBio.00337-19
qPCR protocol	(Zhang et al., 2014)	DOI: 10.1128/MCB.01673-13
RNA-seq library preparation protocol	(Dobrowolski et al., 2019)	DOI: 10.1128/mBio.00337-19
shRNA screen protocol	(Das et al., 2018)	DOI: 10.1073/pnas.1803468115
mSigDB	University of California San Diego	https://www.gsea-msigdb.org/gsea/msigdb/index.jsp
Published primary hematopoietic stem cell quiescence dataset	(Affer et al., 2011)	ACC#: GSE24739
Published iPSC derived microglia HIV infection dataset	(Ryan et al., 2020)	ACC#: GSE143687
Published primary CD4+ T cell ex-vivo HIV latency dataset	(Trypsteen et al., 2019)	ACC#: PRJNA322599
Published primary CD4+ T cell ex-vivo HIV latency	Institute of	ACC#: PRJNA376596

dataset	Microbiology	
Published primary CD4+ T cell activation time course dataset	Johnson & Johnson	ACC#: PRJNA209867
Published SUPT1 HIV infection dataset	(Chang et al., 2011)	ACC#: PRJNA167037
Published SUPT1 HIV infection dataset	(Peng et al., 2014)	ACC#: PRJNA234297
Published H9 HIV infection dataset	(Gupta et al., 2015)	ACC#: PRJNA289529
Published Jurkat HIV infection dataset	Lady Davis Institute for Medical Research	ACC#: PRJNA265857
Published STCO1 and CH077 HIV infection dataset	(Langer et al., 2019)	ACC#: PRJNA482835
Published primary CD4+ T cell HIV infection dataset	University of Pennsylvania	ACC#: PRJNA277687
Gencode release 27	Wellcome Sanger Institute	https://www.gencodegenes.org/human/release_27.html
HIV reporter virus protocol	(Dobrowolski et al., 2019)	DOI: 10.1128/mBio.00337-19
Transcriptome of human primary CD4+ T cells before and after HIV infection, quiescence and reactivation	Sequence Read Archive	SRP145508
Experimental Models: Cell Lines		
Jurkat cells	ATCC	Cat#TIB-152
Oligonucleotides		
Scrambled Negative Control DsiRNA	IDT	Cat#51-01-19-08
TEX 615 Transfection Control DsiRNA	IDT	Cat#51-01-20-21
MYC targeting dsiRNA #1	This paper	5'-AUCAUUGAGCCAAAUCUUAAAAAAA 5'-UUUUUUUAAGAUUUGGCUCAAUGAUAU
MYC targeting dsiRNA #2	This paper	5'-CGACGAGACCUUCAUCAAAAACATC

		5'-GAUGUUUUUGAUGAAGGUCUCGUCGUC
KLF2 targeting dsiRNA #1	This paper	5'-GUGCAAUAAUUUAAGUGG<br>5'-GAAGAUGCCACUUAAAUU
KLF2 targeting dsiRNA #2	This paper	5'-CGAGGCUUGUGAUGCCU<br>5'-UUCUCACAAGGCAUCACA
Barcoded Bead SeqB	ChemGenes	Macosko-2011-10B
Software and Algorithms		
Fastqc	Babraham Bioinformatics	https://www.bioinformatics.babraham.ac.uk/projects/fastqc/
Gene Set Enrichment Analysis (GSEA) package	University of California San Diego	https://www.gsea-msigdb.org/gsea/index.jsp
Trim Galore	Babraham Bioinformatics	https://www.bioinformatics.babraham.ac.uk/projects/trim_galore/
STAR version 2.7.2b	(Dobin and Gingeras, 2015)	https://github.com/alexdobin/STAR
htseq-count	(Anders et al., 2015)	https://htseq.readthedocs.io/en/release_0.11.1/install.html
Kallisto version 0.43.1	(Bray et al., 2016)	https://pachterlab.github.io/kallisto/download
Sleuth	(Pimentel et al., 2017)	https://pachterlab.github.io/sleuth/download
Scanpy suite of packages	(Wolf et al, 2018)	https://scanpy.readthedocs.io/en/stable/
edgeR	(Robinson et al., 2010)	https://bioconductor.org/packages/release/bioc/html/edgeR.html
