# Supplementary figures and images for: HIV infection reprogrammes CD4+ T cells for quiescence and entry into proviral latency
Source: Nat Microbiol. 2025 Sep 26;10(10):2454–71. doi: 10.1038/s41564-025-02128-y (PMC12488490; doi:10.1038/s41564-025-02128-y)

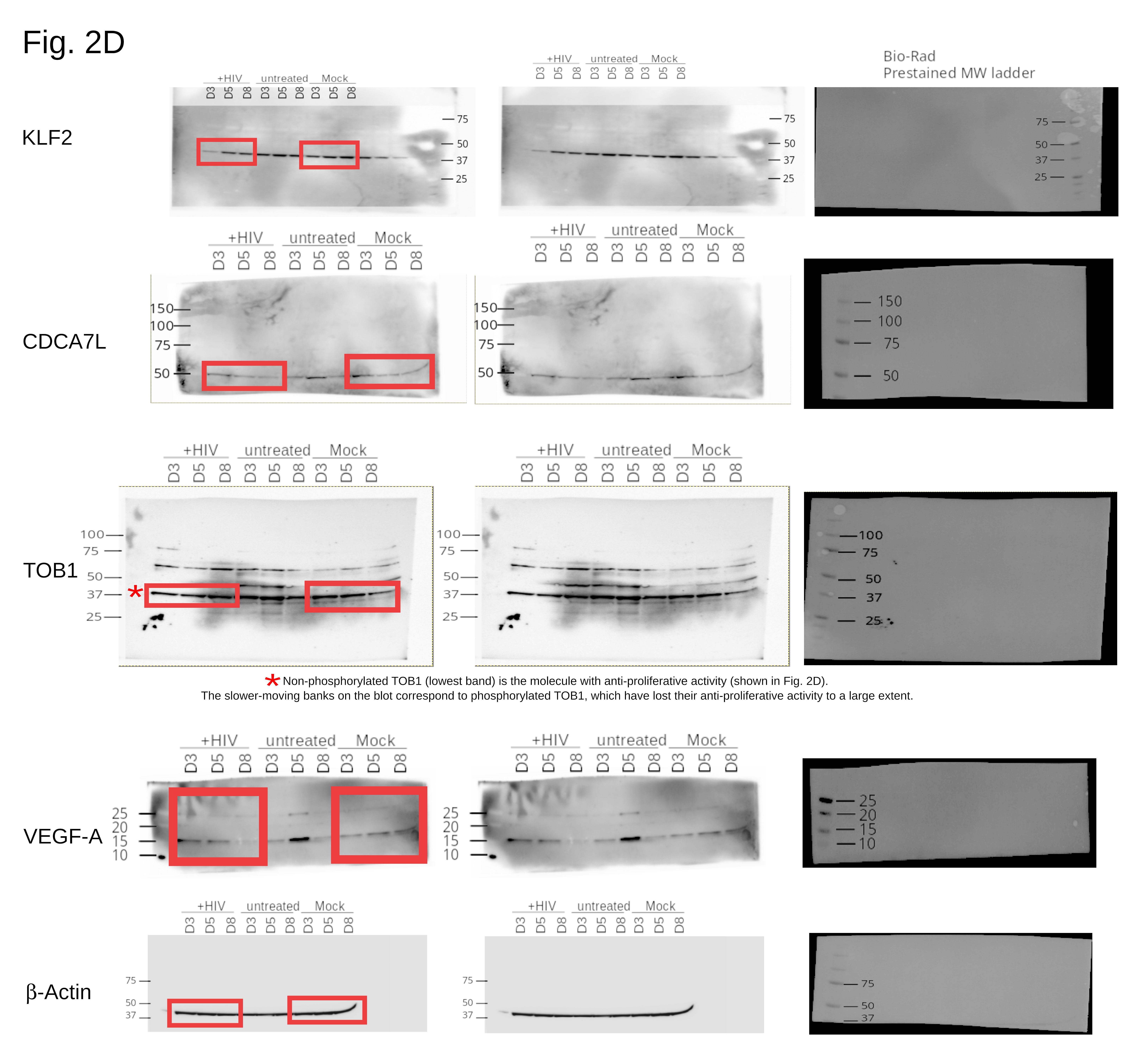

Supplement: Supplementary file 6 — Unprocessed western blots. [file 41564_2025_2128_MOESM6_ESM.jpg]

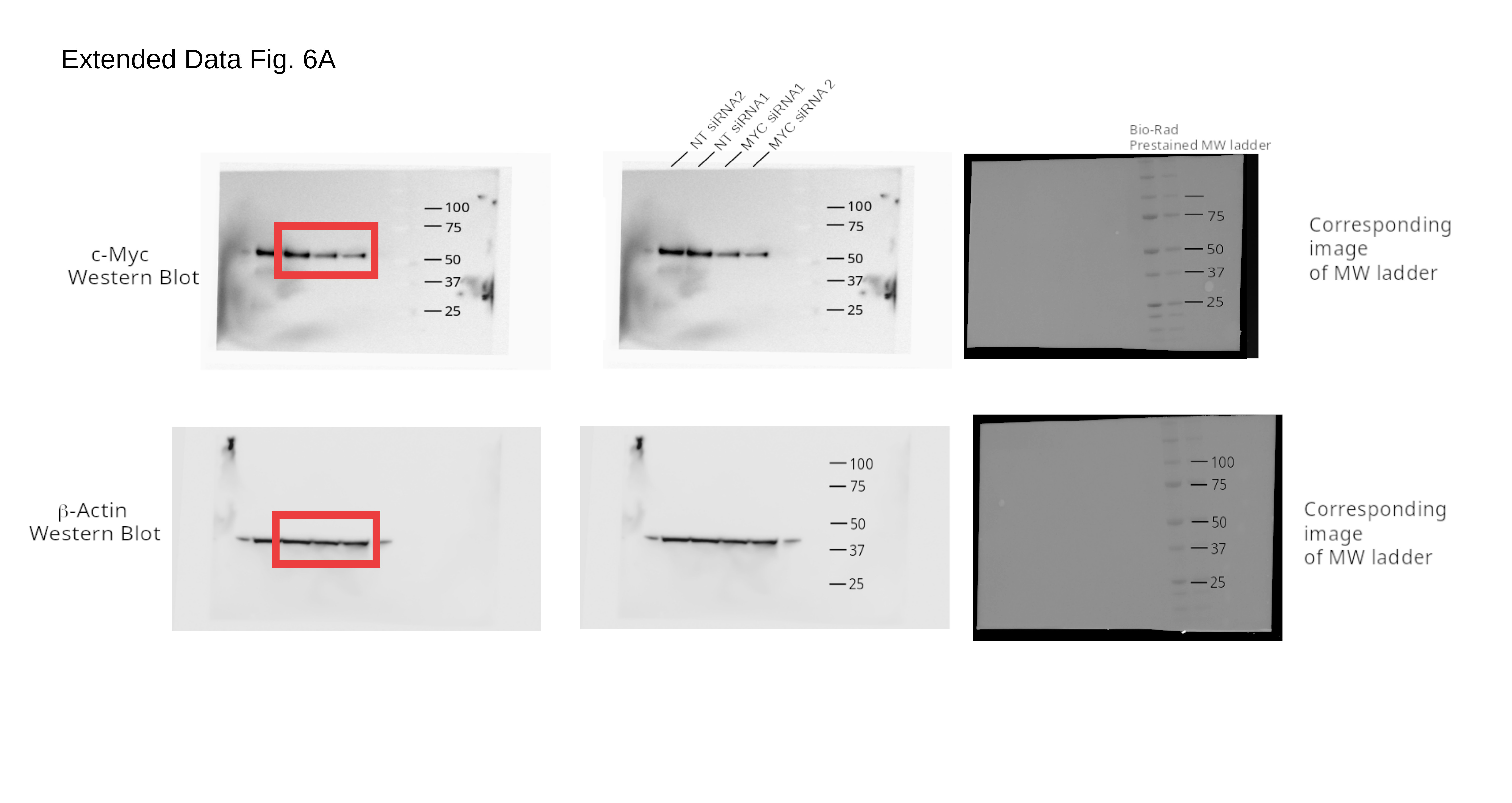

Supplement: Supplementary file 16 — Unprocessed western blots. [file 41564_2025_2128_MOESM16_ESM.jpg]

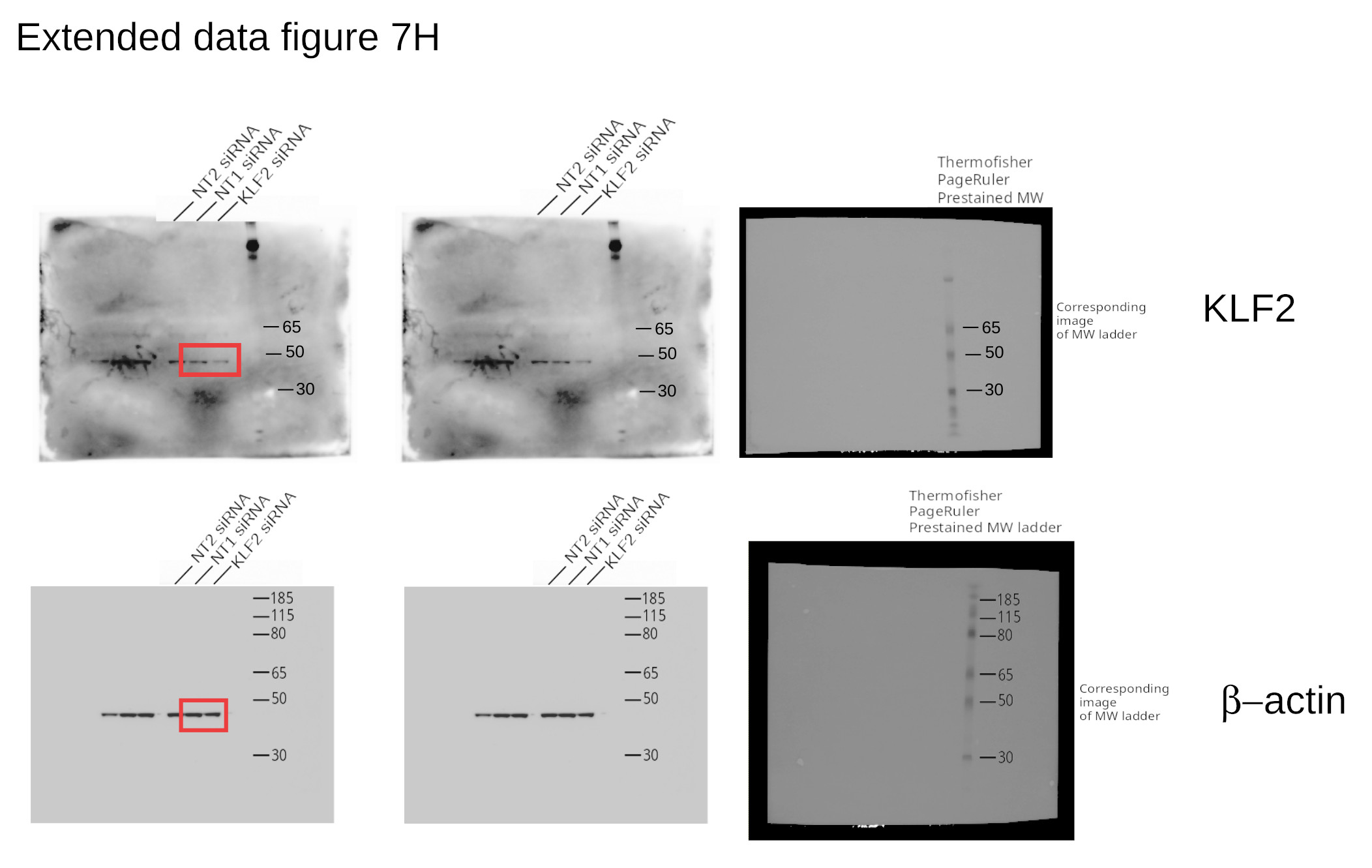

Supplement: Supplementary file 18 — Unprocessed western blots. [file 41564_2025_2128_MOESM18_ESM.jpg]
